# Soft-X-ray momentum microscopy of nonlinear magnon interactions

**DOI:** 10.1038/s41567-026-03318-z

**Published:** 2026-06-05

**Authors:** Steffen Wittrock, Christopher Klose, Salvatore Perna, Korbinian Baumgaertl, Andrea Mucchietto, Michael Schneider, Josefin Fuchs, Victor Deinhart, Tamer Karaman, Dirk Grundler, Stefan Eisebitt, Bastian Pfau, Daniel Schick

**Affiliations:** 1 Max-Born-Institut für Nichtlineare Optik und Kurzzeitspektroskopie, Berlin, Germany; 2 Helmholtz-Zentrum Berlin für Materialien und Energie GmbH, Berlin, Germany; 3 Dipartimento di Ingegneria Elettrica e delle Tecnologie dell’Informazione (DIETI), Università degli Studi di Napoli Federico II, Naples, Italy; 4 Laboratory of Nanoscale Magnetic Materials and Magnonics, Institute of Materials, École Polytechnique Fédérale de Lausanne (EPFL), Lausanne, Switzerland; 5 Institute of Electrical and Micro Engineering, École Polytechnique Fédérale de Lausanne, Lausanne, Switzerland; 6 Institut für Physik und Astronomie, Technische Universität Berlin, Berlin, Germany

**Keywords:** Imaging techniques, Magnetic devices, Spintronics, Imaging techniques, Magnetic properties and materials

## Abstract

Magnons are quantized collective excitations of long-range ordered spins. At nanometre wavelengths, exchange interactions increasingly govern their dynamics, giving rise to a largely unexplored regime of couplings between magnons and other quasi-particles; however, detecting such short-wavelength spin waves has remained a key experimental challenge. Here we introduce magnon momentum microscopy—a quasi-elastic, resonant magnetic soft-X-ray scattering technique that directly images magnon populations across two-dimensional momentum space. Owing to its remarkable sensitivity, it can capture nonlinear magnon interactions over large regions of the dispersion plane. We apply magnon momentum microscopy to the prototypical magnonic material yttrium iron garnet and reveal a rich variety of previously unobserved nonlinear magnon interactions. With its element specificity and bulk sensitivity, as well as intrinsic access to nanometre-scale wavelengths without frequency limitation, this technique establishes a powerful and versatile platform for exploring short-wavelength and nonlinear magnonics.

## Abstract (web summary)

Short-wavelength magnons and their couplings are difficult to detect, limiting studies of nanoscale spin dynamics. Now a method using soft X-rays to image magnon momentum captures their nonlinear interactions with nanometre-scale sensitivity.

## Main

Spin waves and their quasi-particles, magnons, provide a versatile platform for exploring alternative concepts for wave-based information processing beyond conventional complementary metal-oxide-semiconductor (CMOS) technology^1^. In particular, nonlinear magnonics has emerged as a promising domain for realizing computing schemes that exploit the intrinsic nonlinearity of magnon interactions^2–4^. As the magnon wavelength decreases, short-range exchange interactions begin to dominate. In the prototypical magnonic material yttrium iron garnet (YIG), this crossover typically occurs for wavelengths below 100 nm. A major challenge in accessing this largely unexplored regime is the ability to reliably excite and detect such short-wavelength modes^5^.

The excitation of magnons in the sub-100-nm range has been recently demonstrated by the exploitation of spin-torque architectures^6,7^ and spin textures^8,9^ as spin-wave emitters. Alternatively, magnonic grating couplers and ferromagnetic coplanar waveguides can reach the sub-100-nm-wavelength regime by direct electrical microwave excitation of spin waves^10–13^.

On the detection side, accessing such high magnon frequencies or wave vectors remains challenging and is a relevant and topical area of research. Electronic techniques, commonly based on microwave absorption^14^, the spin-Hall effect^15^, spin-wave-transmission^16^ or magnetoresistive detection^17^, have emerged as reliable tools providing information on the frequency-dependent magnon amplitude in the gigahertz regime. Light, however, can be used as a more versatile probe of magnons. Unlike electronic detection, optical methods do not rely on patterned antennas, contacts or Hall structures, allowing flexible scattering and spectroscopy geometries under largely unconstrained sample conditions. Generally, the interaction of photons with magnons can be described quantum-mechanically by the creation/annihilation of magnons in a Stokes/anti-Stokes process. Owing to the conservation of energy and momentum, the scattered photons experience a frequency and momentum transfer from the spin wave (SW) of *f*_f_ = *f*_i_ ∓ *f*_SW_ and **k**_f_ = **k**_i_ ∓ **k**_SW_, respectively, with frequency *f* and wave vector **k** and indices denoting the photon’s initial (i) and final (f) states. Brillouin light scattering in the optical range has been a very successful tool exploiting this inelastic scattering approach in frequency space for magnons up to the few-gigahertz range with momentum and spatial resolution^18–20^.

To truly access the sub-100-nm regime, probing by short-wavelength X-rays is essential. Pioneering techniques such as resonant inelastic X-ray scattering (RIXS)^21,22^ and scanning transmission X-ray microscopy (STXM)^9^ have enabled notable insights, the former in frequency and momentum space, the latter in real space and time domain. However, the direct detection of nonlinear interactions of sub-100-nm magnons across the entire dispersion plane remains a critical challenge. Existing methods, although powerful within their specific domains, face fundamental constraints in sensitivity, efficiency and accessible phase space, leaving a substantial detection gap in magnonics where key dynamic processes unfold unseen. To bridge this gap, we here present the development of magnon momentum microscopy (MMM), an advanced X-ray technique to directly image magnon populations in two-dimensional momentum space. We demonstrate the unique capabilities of this approach by our experimental observation of nonlinear magnon–magnon scattering in the exchange-dominated regime, opening a new window into the exploration of magnon dynamics beyond previous limitations.

## Magnon momentum microscopy

**Fig. 1:**
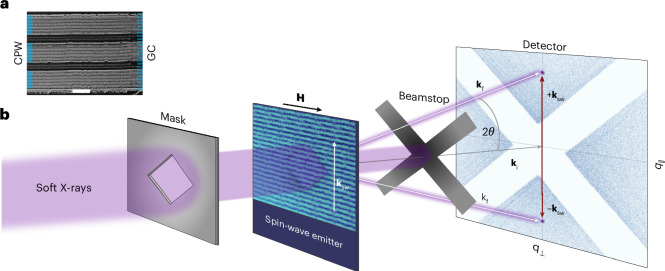
Soft-X-ray MMM. **a**, Scanning electron microscopy image of the spin-wave emitter, consisting of a coplanar waveguide (CPW) and thin permalloy stripes, forming the grating coupler (GC), as indicated by the blue areas. White scalebar, 2 μm. **b**, Schematics of the soft-X-ray scattering geometry. Plane-wave magnons in Damon–Eshbach configuration (*μ*_0_**H**⊥**k**_SW_) propagate away from the spin-wave emitter, as indicated by the bluish, out-of-plane magnetization contrast. Quasi-elastic, resonant magnetic scattering with the magnons results in plus and minus first-order diffraction peaks on the detector, revealing the magnon wave vector, **k**_SW_, directly in momentum space.

In this work, we study magnons down to the sub-100-nm regime by directly accessing their wave vectors. We do so by using quasi-elastic resonant magnetic soft-X-ray scattering as a highly sensitive and effective tool to probe magnons in momentum space. In analogy to recent works on X-ray scattering with temporally coherent phonons^23–26^, we neglect the small energy transfer between the magnons (sub-electron volt) and soft-X-ray photons (hundreds of electron volts), which in a classical picture stems from a Doppler shift of the moving spin wave. Accordingly, we can describe the spin waves as a quasi-static periodic magnetic modulation during the interaction with a soft-X-ray photon^27^. This modulation effectively forms an absorption grating when tuning the photon energy to electronic resonances exhibiting an X-ray magnetic circular dichroism (XMCD)^28^. This concept of MMM is sketched in Fig. 1b for probing propagating spin waves via soft-X-ray scattering. The requirements for this soft-X-ray experiment are modest: a moderate beam focus of tens of micrometres is sufficient. Crucially, the photon energy must match a magnetically sensitive absorption edge to achieve high scattering contrast. Although this absorption contrast relies on a circular dichroism, the magnetic scattering does not require a defined X-ray polarization^28,29^. In a simple transmission scheme, a beamstop prevents the direct beam from hitting the two-dimensional soft-X-ray detector, and a proximity mask defines the probed area of interest and avoids any topographic scattering, resulting in negligible background scattering. For spin waves with a well-defined wave vector **k**_SW_ (with wavelength *λ*_SW_ = 2π/∣**k**_SW_∣), magnetic diffraction peaks of first order emerge in the scattering pattern at **q** = ±**k**_SW_, with **q** being the scattering vector. This simple scattering relation forms the basis of our momentum microscopy image.

To demonstrate and benchmark the capabilities of MMM, we excite magnons down to sub-100 nm by coupling a microwave-generated magnetic field (with radio frequency (RF), *f*_RF_) from a coplanar waveguide to an underlying grating coupler structure, integrated onto a 100-nm-thick YIG film on a gadolinium gallium garnet (GGG) substrate^12,13^, as pictured in Fig. 1a. Some of us recently demonstrated the feasibility of this approach for the generation of propagating sub-100-nm magnons at frequencies up to ~8 GHz by STXM^12^, using a sister sample of the one investigated here. The spin-wave excitation is entirely performed in the Damon–Eshbach configuration, that is, the magnetic field is applied parallel to the coplanar waveguide and grating coupler structures (Fig. 1b). In consequence, the propagation direction of the excited magnons is expected to be purely perpendicular to the field, that is, **k**_SW_⊥*μ*_0_**H**, and the momentum transfer to the soft X-rays occurs along the *q*_∥_ direction. More details on the experimental setup and sample are given in the Methods.

**Extended Data Fig. 1:**
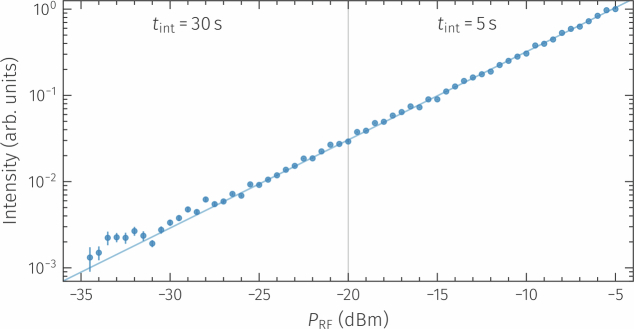
Normalised intensity of the 4*G*-scattering peak at 9.00 GHz for increasing excitation power, *P*_RF_, benchmarking the sensitivity of the magnon momentum microscopy (MMM) technique. The signal-to-noise ratio enables detection of the scattering signal down to *P*_RF_ = − 34 dBm at reasonable acquisition times of 30 s for a single MMM image. For *P*_RF_ > − 20 dBm, the integration time is reduced to only 5 s. Intensity values are obtained from Gaussian peak fits; error bars denote the 1*σ* uncertainties derived from the covariance matrix of the fit. The signal follows the expected exponential behaviour (solid blue line).

The quasi-background-free detection of magnetic scattering with the MMM technique enables unparalleled sensitivity up to high magnon wave vectors. We observe a distinguishable diffraction signal down to an excitation power of at least −34 dBm at an integration time of *t*_int_ = 30 s (Extended Data Fig. 1). For comparison, more than 1,000 times more microwave power (−3 dBm) was required to properly distinguish the spin-wave signal from background noise in STXM^12^ from a sister sample at reasonable integration times.

Direct access to the intensity and wave vector of magnons down to sub-100-nm wavelength within only seconds of integration time is the first main achievement of this work. In the following, we demonstrate that the strength of our MMM concept stretches beyond simple diffraction from well-defined magnons, revealing nonlinear processes uncovered by the two-dimensional accessibility of momentum space.

## Nonlinear magnon processes in two-dimensional momentum space

**Fig. 2:**
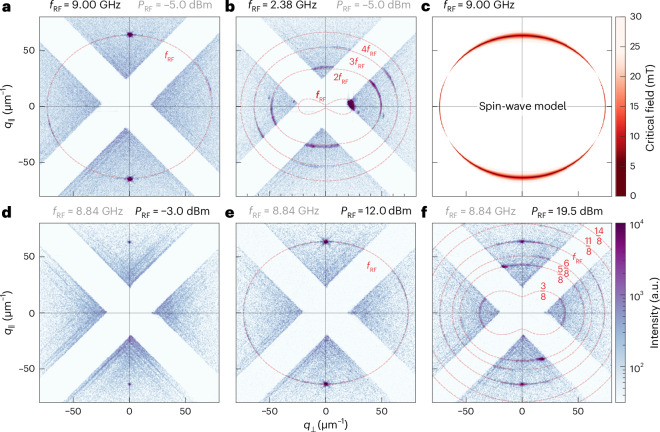
Imaging nonlinear magnon processes in momentum space. **a**, Elliptical dispersion ring resulting from nonlinear magnon–magnon scattering of the directly excited Damon–Eshbach spin waves into modes of arbitrary propagation direction at *f*_RF_ = 9.00 GHz. **b**, Higher harmonics and mode redistribution at *f*_RF_ = 2.38 GHz; the dashed red lines indicate theoretical dispersion curves for *f*_SW_ = *nf*_RF_. **c**, Calculated RF critical field for nonlinear scattering from the spin-wave model, evaluated for the excitation power corresponding to −5.0 dBm used in **a** and **b**. **d–f**, Power-dependent transition at *f*_RF_ = 8.84 GHz (bias field *μ*_0_*H* = 20 mT deviating from other shown measurements) from linear excitation (**d**) to a nonlinearly excited elliptical ring (**e**) and higher/fractional harmonics as indicated (**f**). The dashed red lines represent theoretical dispersion iso-frequency curves for the most prominent harmonics. **a**, **b** and **d**–**f** share the intensity scale.

**Extended Data Fig. 2:**
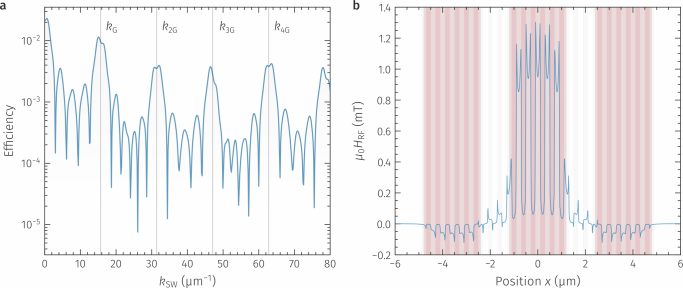
Calculated magnon excitation of the coplanar waveguide and grating coupler. **a** Excitation efficiency of the coplanar waveguide and grating coupler. The calculation is performed by Fourier transformation of the simulated RF in-plane Oersted fields upon microwave excitation. The coplanar waveguide efficiently transfers a wave vector *k*_1_ = 0.85 µm^−1^ to the YIG film^12^. The efficiently transferred larger wave vectors mainly result from the grating coupler pattern. Its natural wave vector is *G* = 2*π*/*a* = 15.7 µm^−1^, along with the higher orders 2*G*, 3*G*, and 4*G*. **b** Magnetic excitation field distribution below the coplanar waveguide (red areas) and grating coupler (grey areas).

The excitation efficiency of magnons by the electronically driven coplanar waveguide and grating coupler structure strongly depends on the driving frequency through the corresponding geometrically favoured wave vectors^12^ (see Extended Data Fig. 2a for excitation spectrum). Hence, at frequencies where the excitation efficiency is high, elevated magnon populations are generated, which foster pronounced nonlinear magnon interactions^30^. In Fig. 2a,b, we show the MMM images at the exact same magnetic field and excitation power as shown in Fig. 1b (*f*_RF_ = 8.68 GHz) but for two distinct excitation frequencies. We provide a movie of the dataset containing the entire frequency sweep in the Supplementary Information. At high frequencies around *f*_RF_ = 9.00 GHz, mainly involving a higher grating coupler mode, the two anticipated diffraction peaks in the *q*_∥_ direction are accompanied by a distinct elliptical scattering ring (Fig. 2a). This ring clearly indicates the population of magnon modes across all directions of propagation. Remarkably, this effect comprises that the directly excited Damon–Eshbach mode along *q*_∥_ ≈ 64 μm^−1^ (*λ*_SW_ ≈ 98 nm) is also scattered into backward-volume magnon modes of larger wave vector along *q*_⊥_ ≈ 73 μm^−1^ (*λ*_SW_ ≈ 86 nm).

To elucidate the underlying nonlinear magnon dynamics, we develop the spin-wave model^30^, detailed in Supplementary Section 1. Our analysis reveals that the mechanism responsible for populating magnon modes across all directions is a spin-wave parametric resonance driven by four-magnon scattering. In this process, two directly excited Damon–Eshbach modes couple to two secondary magnons of arbitrary wave vector ±**k**_SW_. The coupling strength is proportional to the population of the directly excited modes. Parametric excitation becomes possible only when the population of the Damon–Eshbach modes reaches a threshold value corresponding to a threshold RF field (or power) amplitude. The critical field for the parametric excitation-threshold field distribution in momentum space can be derived from the spin-wave model (Supplementary Section 3.2) and is presented in Fig. 2c for an excitation frequency of *f*_RF_ = 9.00 GHz. Importantly, the observed parametric-instability mechanism is fundamentally distinct from the commonly invoked Suhl instability^30^, in which the uniform mode (*k*_SW_ = 0) parametrically excites spin-wave modes with *k*_SW_ ≠ 0. To our knowledge, this represents the first direct observation of an omnidirectional magnon population arising from such a four-magnon parametric process. The calculations of the critical RF field amplitude for parametric excitation reveal that, for the RF power values used in the experiments, only spin-wave modes satisfying the resonance condition *f*_SW_ = *f*_RF_ can be parametrically excited, implying that all magnons on the elliptical diffraction ring in Fig. 2a oscillate at the same frequency. Furthermore, the magnetization deflection angle at the parametric-instability threshold remains small (${\theta }_{\max }\approx {2}^{\circ }$ at *f*_RF_ = 2.38 GHz and ${\theta }_{\max }\approx 1{0}^{\circ }$ at *f*_RF_ = 9.00 GHz; Supplementary Section 3.6). MMM can access the entire magnon dispersion plane in a single acquired image, and we find near-perfect match between the theoretical direction-dependent spin-wave dispersion (dashed red lines in Fig. 2) and the scattering ellipse of the nonlinearly formed modes. Details on the theoretical calculations are provided in the Methods.

We now turn the attention to lower excitation frequencies (*f*_RF_ = 2.38 GHz), where the excitation efficiency is even larger due to the direct coupling of the field generated by the coplanar waveguide to the spin waves (without involving the grating coupler conversion). The corresponding MMM image is shown in Fig. 2b and reveals the nonlinear generation of higher-order modes of similar elliptical shape as in Fig. 2a. These additional modes correspond to higher harmonics of the fundamental mode *f*_SW_ = *nf*_RF_ (*n* = 1, 2, 3, 4) as indicated by the comparison to the theoretical dispersion rings (Fig. 2b, dashed red lines). In this low-frequency, long-wavelength regime, the intense fundamental (first order) almost entirely disappears behind the beamstop due to the optimization of the detection geometry for larger momentum transfer in our experiment. However, we observe that the directional dependence of the second order slightly deviates from the calculated dispersion curve. We attribute this to the high population of these modes and, consequently, the influence of a nonlinear frequency shift^30^. Accordingly, the deviation relaxes for the less intense third and fourth orders, which again show a good match to theory.

We further investigate the threshold behaviour of the nonlinear magnon interactions and acquire MMM images of an extended series of excitation powers. While we again provide the image series as a movie in the Supplementary Information, Fig. 2d–f pictures selected snapshots of a clear hierarchy of nonlinear effects in the exchange-dominated magnon regime. At lower power, single diffraction peaks are detected, reflecting the linear propagation of the excited spin wave. As the power increases, a fundamental dispersion ring forms, followed by additional parametric processes that generate spin waves at fractional harmonics of the fundamental frequency (${f}_{{\rm{SW}}}=\frac{m}{8}{f}_{{\rm{RF}}}$, with *m* = 3 to 14, except for *m* = 4 and 10). The appearance of these modes highlights the deeply nonlinear character of the strongly driven system. While a detailed theoretical understanding of these fractional harmonics is beyond the scope of this study, they underscore the rich information content accessible with MMM and remain a topic for future investigation.

## High-resolution mapping of the spin-wave dispersion

**Fig. 3:**
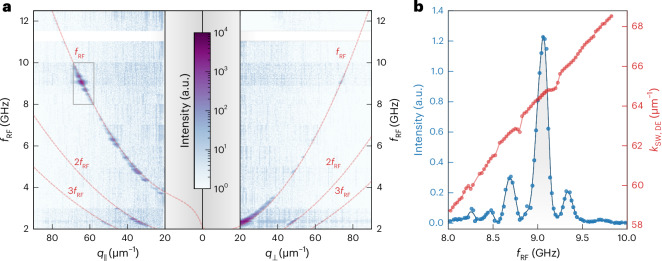
Extracted magnon dispersions. **a**, Dispersion along *q*_∥_ and *q*_⊥_, corresponding to the Damon–Eshbach (DE) and the backward-volume spin-wave modes, respectively. The backward-volume mode arises from nonlinear scattering. The dashed red lines indicate theoretical dispersions for *f*_SW_ = *nf*_RF_ with *n* = 1, 2, 3. **b**, A zoom into the DE dispersion at high frequencies (region marked in **a**), showing intensity maxima and discrete wave vector jumps due to the grating coupler geometry. The lines are guides to the eye.

Determining the spin-wave dispersion relation at submicrometre wavelengths is an experimental challenge, as traditional methods typically fail to capture the magnonic behaviour in this regime. By contrast, MMM directly accesses reciprocal space, naturally enabling the dispersion to be resolved with an unprecedented combination of precision and range. In Fig. 3a, we extract the experimental dispersion curve directly from the extended frequency series, with representative snapshots presented in Fig. 2a,b.

The dispersion for a given propagation direction is readily obtained from directional lineouts of the diffraction data. As the magnon generation is not limited to the primarily excited Damon–Eshbach mode, the nonlinear formation of the fundamental dispersion ring allows access to arbitrary momentum directions. We show the dispersion of the prominent Damon–Eshbach mode along *q*_∥_ (left) and of the backward-volume mode along *q*_⊥_ (right) in Fig. 3a. The data cover a broad range of the dispersion, clearly revealing its general parabolic form (${f}_{{\rm{SW}}}\propto {k}_{{\rm{SW}}}^{2}$), a hallmark of the exchange-dominated nature of the magnons. The theoretical dispersion curves (Fig. 3a, dashed red lines) show a very high agreement with the experimental data for the fundamental as well as the second and third harmonics. Note that the higher-harmonic branches appear at lower frequencies than the fundamental because the dispersion in Fig. 3a is shown as a function of the excitation frequency *f*_RF_. The actual magnon frequencies, however, obey *f*_SW_ = *nf*_RF_, and MMM measures the corresponding wave vectors.

**Extended Data Fig. 3:**
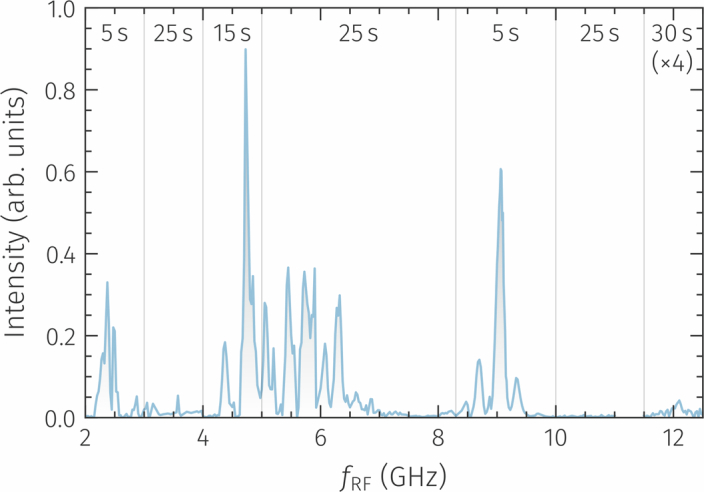
Excitation spectrum. Scattering intensities over the entire frequency range *f*_RF_ at an applied field of *μ*_0_*H* = 30 mT for the Damon-Eshbach direction along *q*_∥_ only. Hence, in the case of nonlinear mode redistribution and ring formation (see Fig. 2), the plotted intensity does not represent the full excitation efficiency. Intensities were normalized to the acquisition time as indicated. For frequencies below 3.30 GHz, the data correspond to second-order scattering, while the first order could be directly analysed for higher frequencies. The high-frequency mode around 12.1 GHz is additionally multiplied by a factor of four for better visibility. Principal peaks at 2.40 GHz, 4.70 GHz, and 9.10 GHz correspond to *k* = *k*_1_, 2*G*, and 4*G* modes, respectively.

As noted earlier, the excitation scheme of coplanar waveguide and grating coupler preferentially generates magnons with specific wave vectors, defined by the grating geometry^12,31^. The resulting scattering intensities, which correspond to the spin-wave intensity, directly reflect this wave-vector-dependent efficiency. As a result, the Damon–Eshbach dispersion curve shows pronounced intensity variations, with several principal intensity peaks, corresponding to favoured wave vectors, such as the fundamental *k*_1_-mode of the coplanar waveguide at 2.4 GHz and the second-order mode of the grating coupler (2*G*) at 4.7 GHz (ref. ^12^). We provide an extended frequency-dependent lineout as Extended Data Fig. 3.

In Fig. 3b, we focus on the frequency band related to the efficient excitation of large wave vectors mediated through the grating coupler periodicity *a* = 400 nm. The dominant diffraction peak of the Damon–Eshbach (DE) mode corresponds to *k*_SW,DE_ = 4*G* = 8π/*a*, with weaker peaks arising from modulations of 4*G* with other coplanar waveguide-preferred wave vectors. The extracted wave vectors (red curve) show a discontinuous frequency dependence, where spin waves are predominantly excited at discrete wave vectors, reflecting confinement by the excitation geometry. Transitions between these preferred values are marked by abrupt jumps and, hence, slight local bending of the dispersion, indicating pulling effects toward geometrically favoured modes. This level of detail, observed in a planar film region micrometres away from the emitter, highlights the sensitivity and momentum resolution of MMM.

## Discussion

A central motivation for the development of MMM is to enable the direct detection of magnons with nanometre-scale wavelengths entering the exchange-dominated regime—a domain largely inaccessible with existing techniques. In this first demonstration of MMM, we directly excite and detect magnon wave vectors up to *q*_∥_ = 79.2 μm^−1^ (*f*_RF_ = 12.1 GHz, *λ*_SW_ ≈ 79 nm) and observe even higher wave vectors for nonlinearly excited magnon modes reaching *q*_∥_ = 93.8 μm^−1^ (*λ*_SW_ ≈ 67 nm), as shown in Fig. 2f. The upper limit for accessible wave vectors in MMM is ultimately set by the overall diffraction limit, specifically, by the wavelength of the scattered soft-X-ray photons, *λ*_ph_ ≈ 1.75 nm, corresponding to 2*k*_ph_ ≈ 7,200 μm^−1^. In our current setup, the maximum detectable wave vector is further limited by the detector geometry, whereas the beamstop obscures features at *q* < 20 μm^−1^. However, these setup parameters are readily adaptable to specific experimental requirements.

Other detection techniques are also pushing into the sub-100-nm regime, but each comes with trade-offs in terms of resolution, sensitivity or data interpretation. Near-field optical approaches, for example, have been proposed to overcome the diffraction limit of conventional magneto-optical Kerr and Brillouin light scattering techniques by using evanescent waves or sharp tips to access the subwavelength regime^20,32^. However, the near-field signal is often highly complex and requires extensive electromagnetic simulations for interpretation. STXM has recently demonstrated access to magnons with wavelengths near 100 nm (refs. ^9,12^). Although powerful in resolving real-space spin dynamics, the sensitivity of STXM to short (and fast) spin waves is inherently limited by its spatial and temporal resolution, which is dictated by the X-ray optics and pulse duration, respectively. RIXS offers access to large parts of the Brillouin zone, probing magnon dispersions up to THz frequencies and Ångström wavelength^21,22^. However, as an energy-domain method, it is limited in detecting low-energy magnons (<100 GHz) requiring sub-millielectron-volt resolution. In addition, RIXS measurements are extremely photon hungry and slow due to inherently weak inelastic scattering cross-sections and losses of dispersive optics.

Magnon probes other than light are often susceptible to variations in the sample environment or rely on stringent external conditions. For example, the resonance frequency of nitrogen-vacancy centres shifts in external magnetic fields^33,34^, complicating measurements. Likewise, the impressive progress achieved by combining scanning transmission electron microscopy and electron energy loss spectroscopy to access antiferromagnetic magnons across the full Brillouin zone with millielectron volt energy resolution^35^ relies on strong magnetic electron lenses, which are difficult to reconcile with most ferromagnetic spin-wave materials.

What sets MMM apart from other approaches is its combination of high sensitivity, rapid acquisition and direct two-dimensional access to momentum space without frequency limitations—all within a relatively simple experimental setting. These capabilities originate from the underlying scattering process, where spin-wave-induced magnetic modulations form an effective absorption grating. Although inelastic processes may contribute^36^, their cross-sections are orders of magnitude weaker than the elastic forward scattering. Operating directly in reciprocal space, MMM bypasses the limitations of inefficient X-ray imaging or dispersion optics, with two-dimensional diffraction patterns that directly encode magnon wave vectors and intensities, eliminating the need for complex reconstruction or modelling.

The efficiency, sensitivity and direct momentum-space access of MMM enable the study of spin-wave phenomena far beyond the reach of existing techniques, most notably nonlinear magnon interactions at nanometre wavelengths. Remarkably, we observe such nonlinear effects in YIG, a textbook model system in magnonics. Specifically, we directly detect a four-magnon scattering process that parametrically redistributes the Damon–Eshbach mode into an omnidirectional population of secondary magnons, forming a characteristic elliptical ring in momentum space. To interpret this behaviour, we developed a spin-wave model that reformulates the classical Landau–Lifshitz description in terms of spin-wave amplitudes, enabling a clear analysis of the nonlinear interaction terms. The model captures a parametric-instability mechanism distinct from conventional Suhl processes and predicts instability thresholds consistent with our observations. That such rich and previously unresolved physics emerges in a prototypical material such as YIG highlights the unique capability of MMM to reveal hidden aspects of magnon dynamics.

## Perspective

MMM offers a powerful new approach for exploring nonlinear spin-wave interactions, mode coupling and wave-vector-resolved scattering processes across a broad range of magnonic systems and geometries. Its high sensitivity, element specificity and direct momentum-space access make it well suited for studying multilayer structures, buried interfaces and engineered magnonic devices. The technique is compatible with a wide variety of magnon excitation schemes and sample environments. MMM can readily be adapted towards material systems with shorter spin-wave propagation lengths than YIG by reducing the soft-X-ray-magnon interaction volume using smaller X-ray apertures or dedicated focussing schemes down to the submicrometre regime. It can be extended to detect magnons up to terahertz frequencies^37,38^ in antiferromagnetic materials by accessing a magnon-induced dynamic net magnetization either by XMCD^39^ or depending on the geometry by exploiting XMLD as a contrast mechanism. Time-resolved implementations using short-pulsed X-ray sources, particularly including laser-based laboratory sources, can provide direct insight into frequency-resolved magnon phenomena on ultrafast timescales^24,26,27^. By enabling direct access to magnon distributions in momentum space, MMM establishes a new experimental paradigm for investigating nonlinear spin-wave dynamics beyond the reach of conventional techniques.

## Methods

### YIG sample

The 100-nm-thick YIG layer is grown on a GGG substrate via liquid-phase epitaxy by Matesy GmbH, Jena, Germany. Onto the YIG film, a 20-nm permalloy (Py, Ni_81_Fe_19_) film is deposited via e-beam evaporation, which is subsequently patterned via e-beam lithography to define the grating couplers in a negative hydrogen silsesquioxane resist. The Py stripes of the grating coupler are chosen to have a width of 200 nm and a periodicity of *a* = 400 nm. The defined grating coupler structures are perpetuated by Ar^+^ ion beam etching, optimized for minimizing the overetching into the YIG film. The coplanar waveguides are fabricated above the grating coupler by using positive polymethylmethacrylate/methylmethacrylate e-beam resist and evaporation of Ti(5)/Cu(110) onto the patterned structure (nanometre thickness in brackets). A scanning electron microscopy image of the fabricated structures is shown in Fig. 1a.

**Extended Data Fig. 4:**
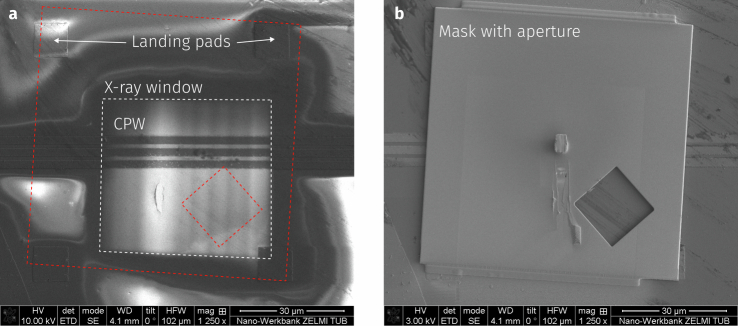
Scanning electron microscopy (SEM) images of the sample layout. **a**, Sample area with X-ray-transparent window before attaching the mask. The image is recorded with 10-keV electrons to penetrate through the window, which is crossed by the coplanar waveguide. Red dashed lines indicate the latter position of the mask and the aperture. Four landing pads were used to slightly elevate the mask above the coplanar waveguide. Bright areas at the rim of the image are caused by charging of the insulating YIG layer. **b**, Same sample area as in a after attaching the mask. The image was recorded using 3-keV electrons to highlight surface details.

X-ray transparency of the sample is achieved by thinning the GGG substrate. This process involves initially mechanically thinning the substrate from its backside, followed by local refinement using focused Ga^+^ ion beam etching^40^. To restrict the region probed by the X-ray beam, we used a square-shaped mask that defines the illuminated area on the sample (Extended Data Fig. 4). Its main purpose is to confine the illumination to a topographically uniform region, thereby suppressing unwanted topographic (charge) scattering from surrounding structures such as the coplanar waveguide or grating coupler, which would otherwise obscure the magnetic scattering from the spin waves. The mask was fabricated from a Cr/Au multilayer (total thickness of ~1 μm) deposited on a SiN membrane, into which a 16 × 16-μm^2^ square aperture was milled using a Ga^+^ focused ion beam. It was then transferred onto the front side of the sample and attached by focused ion beam-assisted Pt deposition. The aperture defines the illuminated detection region in the bare YIG, with its centre located approximately 11 μm away from the coplanar waveguide. The SiN support electrically insulates the metallic mask from the coplanar waveguide, preventing any shorting.

Introducing such an aperture inevitably produces additional scattering from the mask edges. To minimize this effect, the square-shaped mask was designed such that the edge-induced scattering occurs primarily along two perpendicular directions defined by the mask edges. These scattering streaks are effectively blocked by a cross-shaped beamstop placed in the detection path. For this reason, the mask was intentionally rotated by 45^∘^ with respect to the expected Damon–Eshbach magnon wave vector direction *q*_∥_. This geometry ensures that the regions affected by mask-edge scattering coincide with the beamstop coverage and are thus blanked out in the MMM images, whereas the magnetic scattering signal remains unaffected. In future implementations of the method, the mask may be substituted by a well-focused and well-defined X-ray beam properly positioned on the sample.

### Experimental setup

We used resonant magnetic scattering as depicted in Fig. 1b and described in the main text. The experiments were conducted with our mobile MAXI endstation at the beamline P04 of the synchrotron-radiation facility PETRA III at DESY, Hamburg and at the beamline UE52-SGM of the BESSY II storage ring at HZB, Berlin. The photon energy is tuned to *E*_ph_ = 708 eV, selecting the maximum XMCD contrast at the Fe L_3_ (2*p* → 3*d*) absorption edge of the YIG film. We used circularly polarized X-rays at PETRA III and linear horizontal polarization at BESSY II. The propagating magnons are excited by applying an RF electrical signal to the coplanar waveguide. The microwave power is chosen to be *P*_RF_ = −5.0 dBm if not stated differently. An in-plane magnetic bias field of 30 mT was applied by an electromagnet, directed along the coplanar waveguide in Damon–Eshbach configuration. As an exception, the data shown in Fig. 2d–f were measured at 20 mT. The relative distances between the sample, beamstop and CCD X-ray detector define the accessible wave vectors in the experiment. Here, we choose the sample-to-CCD distance to *z*_0_ = 260 mm, which defines the scattering signal position on the CCD chip through *d* = *k*_SW_ *z*_0_/*k*_i_, with *k*_SW_ the wave number of the magnons, *k*_i_ = 2π*E*_ph_/(*hc*) the wave number of the incident soft X-rays. Adjusting these parameters and the beamstop position allows further flexibility regarding the accessibility of magnon wave vectors.

### Data analysis

The raw data, such as shown in Figs. 1b and 2, are obtained by directly evaluating the CCD images acquired. For each dataset, we subtract a background image, with the RF excitation switched off, from the image containing magnetic information (RF excitation on). The background accounts for the detector offset and remaining topographic scattering. Before subtraction, we apply a subpixel drift correction between both detector images. To obtain the intensity and momentum-space position of the scattering peaks in the two-dimensional scattering plane, they are fitted using two-dimensional Gaussian profiles (used in Fig. 3b and Extended Data Fig. 3). To extract the dispersion from the data (Fig. 3a), we perform lineouts along selected directions in reciprocal space, defined by specific azimuthal angles. These directions can be chosen to correspond to the Damon–Eshbach (along *q*_∥_) and backward-volume (along *q*_⊥_) spin-wave modes (such as in Fig. 3a) or any other arbitrary orientation. By plotting the scattering intensity along these lineouts as a function of the scattering vector, *q*, we obtain intensity profiles that reflect the spin-wave characteristics.

### Nonlinear spin-wave dynamics

In the MMM images, we observe that above a certain RF power, *P*_RF_, and for a fixed excitation frequency, *f*_RF_ = *ω*_RF_/(2π), spin waves begin to propagate in directions different from that of the directly driven mode. This behaviour indicates the onset of a spin-wave instability, which can be understood by examining the coupled nonlinear dynamics of magnons in the YIG film. In the linear regime, magnon modes are uncoupled (Supplementary Section 2). However, we show that the excitation of spin waves, which are not directly driven by the RF field arises from a nonlinear mechanism—specifically, a parametric resonance. To analyse this, we reformulate the Landau–Lifshitz equation in terms of spin-wave amplitudes, leading to our spin-wave model. A full derivation of the spin-wave model, inspired by the classic work of Suhl^30^, is provided in Supplementary Section 1.

Using the spin-wave model, we first characterize the system’s nonlinear dynamics and then derive the dispersion relation. This analysis reveals that the magnons excited via nonlinear processes, forming the elliptical scattering ring, resonate at the same frequency as the RF field. Among the nonlinear interaction terms in the spin-wave model, we isolate the parametric terms that are resonant with the RF field and involve either the Damon–Eshbach spin waves or the RF mode itself. The concept is that, before the onset of instability, only the Damon–Eshbach spin waves and the RF field exhibit notable amplitudes.

Following an approach similar to Suhl^30^, we derive an expression for the threshold RF field required to trigger the parametric spin-wave instability. The predicted threshold values align well with those used in our experiments. Finally, the distribution of these threshold values across momentum space offers critical insight into the features observed in the MMM images, as discussed later in this section.

#### Dispersion relation

In the case of linear and conservative dynamics, spin-wave normal mode amplitudes evolve independently with a frequency given by the following dispersion relation (Supplementary Section 2):

1 $${\omega }_{k}=\gamma {\mu }_{0}{M}_{{\rm{s}}}\sqrt{\left(H-{N}_{x}+{l}_{\mathrm{ex}}^{2}{k}^{2}+{N}_{k,yy}\right)\left(H-{N}_{x}+{l}_{\mathrm{ex}}^{2}{k}^{2}+{N}_{k,zz}\right)}\,,$$

where *ω*_*k*_ is the natural frequency in dimensional unit for wave vector ${\bf{k}}={k}_{x}\widehat{{\bf{x}}}+{k}_{y}\widehat{{\bf{y}}}\ne {\boldsymbol{0}}$, *γ* is the absolute value of the gyromagnetic ratio, *M*_s_ is the saturation magnetization, *H* = *H*_dc_/*M*_s_ is the uniform, direct current (d.c.) field applied along the equilibrium direction (*x*), *N*_*x*_ is the demagnetizing factor along the *x* direction, ${l}_{{\rm{ex}}}=\sqrt{2{A}_{{\rm{ex}}}/{\mu }_{0}{M}_{{\rm{s}}}^{2}}$ is the exchange length with *A*_ex_ the exchange stiffness and *μ*_0_ the vacuum permeability and *N*_*k*,*yy*_, *N*_*k*,*zz*_ are the components of the Fourier transform of the demagnetizing tensor computed assuming the magnetization uniform along the thickness of the sample. In the next section, *ω*_*k*_ will be indicated as the spin-wave normal mode’s frequency in dimensionless form (normalized to *γM*_s_). The material and experimental parameters used for the theoretical modelling—including the calculation of the magnon dispersion relation (Figs. 2a,b,e,f and 3a) and the threshold field for the parametric instability (Fig. 2c)—are listed in Table 1.

**Table 1:** Values of the experimental and material parameters for the calculation of the directional dispersion relation and the threshold field of the parametric instability

Name	Symbol	Unit	Value
Gyromagnetic ratio	*γ*	Hz T^−1^	2π × 28.024 × 10^9^
Saturation magnetization	*μ*_0_*M*_s_	mT	175
Exchange stiffness	*A*_ex_	pJ/m	3.65
Film thickness	*d*	nm	100
Gilbert damping	*α*	—	1 × 10^−4^

For the dataset used in Figs. 2a,b,c and 3a, the nominal field value of *μ*_0_*H* = 30 mT is reduced to 27 mT for a better curve match in Fig. 3a, a value within the precision range of our electromagnet. For the curves in Fig. 2d,e,f, *μ*_0_*H* = 20 mT is used.

#### Threshold for spin-wave parametric excitation

The spin-wave model contains a variety of nonlinear terms that describe magnon–magnon interactions. However, not all of these terms are relevant for deriving the threshold field for parametric excitation of spin waves that are not directly driven by the RF field. To focus on the relevant dynamics, we derive a simplified model from the spin-wave model, retaining only the nonlinear terms associated with four-magnon scattering processes. These terms are both parametric and resonant with the RF field and allow us to obtain the following expression for the critical RF field, *H*_RF,crit_, required to parametrically excite a spin wave with wave number *k*

2 $${H}_{\mathrm{RF,crit}}={\mu }_{0}{M}_{{\rm{s}}}{\left(\frac{{({\omega }_{\mathrm{RF}}-{\omega }_{k})}^{2}+{\alpha }^{2}{A}_{k}^{2}}{{\left|{\sum }_{k{\prime} \in \{0,{k}_{\mathrm{DE}}\}}\frac{{\zeta }_{kk{\prime} }{\widehat{h}}_{k{\prime} }^{+2}}{{({\omega }_{\mathrm{RF}}-{\omega }_{k{\prime} }-i\alpha {A}_{k{\prime} })}^{2}}\right|}^{2}}\right)}^{\frac{1}{4}}.$$

Here, *k*_DE_ is the wave number of the Damon–Eshbach spin waves, ${\zeta }_{kk{\prime} }$ is a coefficient describing the coupling between the spin wave with wave number *k* and the Damon–Eshbach spin wave with wave number $k{\prime}$, and ${\widehat{h}}_{k{\prime} }^{+}$ is the efficiency of the coplanar waveguide and grating coupler corresponding to the counterclockwise *k*′th component of the RF field. All the quantities between parentheses are dimensionless, whereas the critical field is expressed in millitesla. In Supplementary Section 3, we show that the observed spin-wave parametric instability is connected to the well-known second-order Suhl instability, yet it is distinct and describes a more general case.

The expression of the critical field allows us to derive the threshold field: it sets the minimum critical RF field—and therefore RF power—required to initiate the parametric excitation of omnidirectional spin waves. For experimental comparison, only this threshold value is relevant. A simple but approximate expression for such a value can be obtained from equation (2) by setting the resonance condition: ${\omega }_{k}={\omega }_{\widetilde{k}}={\omega }_{\mathrm{RF}}$ and $k=\widetilde{k}\in \{{k}_{\mathrm{DE}}\}$, that produces the following relation:

3 $${H}_{\mathrm{RF,thr}}=\mathop{\min \,}\limits_{k}{H}_{\mathrm{RF,crit}}={\mu }_{0}{M}_{{\rm{s}}}\left(\frac{{A}_{\widetilde{k}}}{| {B}_{\widetilde{k}}| }\,\frac{{\alpha }^{3/2}{\omega }_{\mathrm{RF}}\sqrt{{A}_{\widetilde{k}}}}{| {\widehat{h}}_{\widetilde{k}}^{+}| \sqrt{H-{N}_{x}}}\right)\,.$$

Above the threshold value of the RF field, nonlinear effects cause the excitation of additional spin waves, and the corresponding threshold values for these modes may be altered by the presence of already excited ones.

#### Experimental implications

In materials such as YIG, characterized by low magnetic damping (*α* ≈ 10^−4^), only Damon–Eshbach spin waves with wave number $k{\prime}$ satisfying ${\omega }_{k{\prime} }\approx {\omega }_{\mathrm{RF}}$ contribute substantially to the denominator in equation (2), unless $| {\widehat{h}}_{k{\prime} }^{+}| \approx \alpha$. From equation (3), it follows that the critical RF field scales proportionally to ${(\alpha {\omega }_{{\rm{RF}}})}^{3/2}$ and inversely with the coplanar waveguide and grating coupler excitation efficiency. A similar relationship has been reported in ref. ^30^.

An order-of-magnitude analysis shows that when the coplanar waveguide and grating coupler efficiency (as shown in Extended Data Fig. 2a) is $| {\widehat{h}}_{k{\prime} }^{+}| \approx 5\times 1{0}^{-3}$, the threshold field is *H*_RF,thr_ ≈ 1 mT, whereas for maximum efficiency (≈ 2 × 10^−2^), the threshold field decreases to *H*_RF,thr_ ≈ 0.01 mT. These values are consistent with the experiments: the threshold power value at *f*_RF_ = 9.00 GHz is *P*_RF_ ≈ −5.0 dBm (Fig. 2a). This power corresponds, according to the COMSOL calculations presented in the Extended Data Fig. 2b, to an RF field amplitude of 1.3 mT. At lower RF frequencies, *f*_RF_ = 2.38 GHz, the same level of RF power excites additional higher-order spin-wave modes of frequencies 2*f*_RF_, 3*f*_RF_ and 4*f*_RF_ (Fig. 2b). The theoretically estimated threshold field (power) is almost two (one) orders of magnitude smaller than the value at *f*_RF_ = 9.00 GHz. This is due to the higher coupling efficiency of the coplanar waveguide at lower wave vectors. Therefore, the excitation of higher-order parametric resonances (*nf*_RF_, *n* = 2, 3, 4) is consistent with the fact that the RF field (power) substantially exceeds the threshold value.

In Supplemental Section 3.6, we present calculations of the maximum magnetization deflection angle ${\theta }_{\max }$ at the threshold RF field for parametric excitation of spin waves. For *f*_RF_ = 2.38 GHz, we find ${\theta }_{\max }\approx {2}^{\circ }$, and for *f*_RF_ = 9.00 GHz, ${\theta }_{\max }\approx 1{0}^{\circ }$. These results indicate that the growth of the Damon–Eshbach spin-wave amplitude is self-limited by the onset of parametric excitation. The small values of ${\theta }_{\max }$ further confirm the consistency of the linear approximation used to describe the Damon–Eshbach spin-wave dynamics below the threshold. In Fig. 2a,b, we observe spin waves at frequencies corresponding to integer multiples of the RF frequency, confirming the excitation of higher-order parametric resonances beyond the primary one. As *ω*_RF_ deviates from the linear spin-wave dispersion, the critical RF field increases sharply, thereby suppressing off-resonant modes. This behaviour is consistent with the experimental observation that, for a fixed RF power above the threshold, the parametrically excited spin waves occupy a narrow region around the *ω*_*k*_ = *ω*_RF_ dispersion curve, with noticeable deviations appearing only at much higher power values.

## Online content

Any methods, additional references, Nature Portfolio reporting summaries, source data, extended data, supplementary information, acknowledgements, peer review information; details of author contributions and competing interests; and statements of data and code availability are available at 10.1038/s41567-026-03318-z.

## Supplementary information

Supplementary Information Supplementary Sections 1–3, including Supplementary Fig. 1.

Peer Review File

Supplementary Video 1 MMM movie for a frequency sweep at constant excitation power *P*_RF_ = −5.0 dBm.

Supplementary Video 2 MMM movie for a power sweep at constant excitation frequency *f*_RF_ = 8.84 GHz.

## Data Availability

The data that support the figures within this study are available via Zenodo at 10.5281/zenodo.19471019 (ref. ^41^).

## Code availability

The code used to generate Figs. 2 and 3 of the main text, Extended Data Figs. 1–3 and Supplementary Videos 1 and 2 is available via Zenodo at 10.5281/zenodo.19471019 (ref. ^41^).
