# Supplementary material for: Soft-X-ray momentum microscopy of nonlinear magnon interactions
Source: Nat Phys. 2026 Jun 5;22(7):1160–5. doi: 10.1038/s41567-026-03318-z (PMC13423880; doi:10.1038/s41567-026-03318-z)
Supplement: Supplementary file 1 — Supplementary Sections 1–3, including Supplementary Fig. 1. [file 41567_2026_3318_MOESM1_ESM.pdf]

nature physics

Supplementary information https://doi.org/10.1038/s41567-026-03318-z

Soft-X-ray momentum microscopy of nonlinear magnon interactions

In the format provided by the authors and unedited

Supplementary Information - *Soft-X-ray momentum microscopy of nonlinear magnon interactions*

S1 Spin-Waves Model

In this Supplementary Information, we develop the spin-wave model (SWM) that will be used to explain the observed nonlinear spin-wave dynamics. The magnetic sample will be assumed uniformly magnetised along x direction due to the presence of a uniform direct current (DC) magnetic field, simply called DC-field in the following, directed in the same direction. The spin-wave dynamics is excited by an radio frequency (RF) field directed always in the plane of the sample but orthogonal to the uniform field, namely the y direction. The magnetisation evolution is described by the Landau-Lifshitz equation [1]:

$$\frac{\partial \mathbf{m}}{\partial t} = -\mathbf{m} \times \mathbf{h}_{\text{eff}} - \alpha\, \mathbf{m} \times (\mathbf{m} \times \mathbf{h}_{\text{eff}}) \ , \tag{1}$$

where $\mathbf{m}(\mathbf{r}, t) = \mathbf{M}(\mathbf{r}, t)/M_{\text{s}}(T)$, with $M_{\text{s}}(T)$ the saturation magnetisation at the temperature T, t is the dimensionless time normalized to $(\gamma M_{\text{s}})^{-1}$, with γ the absolute value of the gyromagnetic ratio, α the damping coefficient, and $\mathbf{h}_{\text{eff}} = \mathbf{H}_{\text{eff}}/M_{\text{s}}(T)$, with $\mathbf{H}_{\text{eff}} = \mathbf{H}_{\text{ex}} + \mathbf{H}_{\text{m}} + \mathbf{H} + \mathbf{H}_{\text{RF}}$ the effective field given by the sum of the exchange field, magnetostatic field, uniform DC field, and the RF field respectively. The magnetisation is assumed to be uniform along the film's thickness (z direction). This fact permits the substitution of the effective field in the Eq. (1) with that one averaged along the thickness. In the following, when we refer to the effective field, its average along the film's thickness is considered. As a first step, let us derive the SWM considering only the conservative magnetisation dynamics. By using the same formalism of Suhl [2], we express the unitary magnetisation components in the following way:

$$\begin{aligned} m_x &= \sqrt{1 - m_y^2 - m_z^2} \,, \\ m_y &= \sum_k m_{y,k}\, e^{j\mathbf{k}\cdot\mathbf{r}} \,, \\ m_z &= \sum_k m_{z,k}\, e^{j\mathbf{k}\cdot\mathbf{r}} \,, \end{aligned} \tag{2}$$

where $k = (k_x, k_y)$ is a multi-index and $\mathbf{k}$ is the corresponding two-dimensional vector defined on the k-space plane, $(m_{x,k}, m_{y,k}, m_{z,k})$ are the Fourier transforms of the unitary magnetisation components, and $\mathbf{r}$ is the in-plane position vector. Let us introduce the kth spin-wave amplitude $a_k = m_{y,k} + im_{z,k}$ and $a_{-k} = m_{y,k} - im_{z,k}$. In this

respect, we can introduce the following auxiliary variables:

$$
\begin{aligned}
m_+ &= m_y + i m_z = \sum_k a_k \, e^{j\mathbf{k}\cdot\mathbf{r}} \,, \\
m_- &= m_y - i m_z = \sum_k a^*_{-k} \, e^{j\mathbf{k}\cdot\mathbf{r}} \,, \\
m_x &= \sqrt{1 - m_+ m_-} \approx 1 - \frac{1}{2} m_+ m_- = 1 - \frac{1}{2} \sum_{k,k'} a_{k'} a^*_{k'-k} \, e^{j\mathbf{k}\cdot\mathbf{r}}
\end{aligned}
\tag{3}
$$

where we made the so-called parabolic approximation in the last equation. The conservative dynamics of the variable m_+ is described by the following relation:

$$
\frac{\partial m_+}{\partial t} = \sum_k \frac{\mathrm{d}a_k}{\mathrm{d}t} \, e^{j\mathbf{k}\cdot\mathbf{r}} = i h_{\mathrm{eff,x}} m_+ - i m_x (h_{\mathrm{eff,y}} + i h_{\mathrm{eff,z}}) \tag{4}
$$

where

$$
\begin{aligned}
h_{\mathrm{eff,y}} + i h_{\mathrm{eff,z}} = &- \frac{(N_y + N_z)}{2} a_0 - \frac{(N_y - N_z)}{2} a_0^* \\
&+ \sum_k e^{-\mathbf{k}\cdot\mathbf{r}} \Bigg[h_{\mathrm{RF},k} - \left(l_{\mathrm{ex}}^2 k^2 + \frac{N_{k,yy} + N_{k,zz}}{2} \right) a_k \\
&\qquad - \frac{N_{k,yy} - N_{k,zz}}{2} a^*_{-k} \\
&\qquad + \frac{N_{k,xy}}{2} \sum_{k'} a_{k'} a^*_{k'-k} \Bigg] \,,
\end{aligned}
\tag{5}
$$

and

$$
\begin{aligned}
h_{\mathrm{eff,x}} = H - N_x \left(1 - \frac{1}{2} \sum_k |a_k|^2 \right) - \sum_k e^{i\mathbf{k}\cdot\mathbf{r}} \frac{N_{k,xy}}{2} \left(a_k + a^*_{-k} \right) \\
+ \frac{1}{2} \sum_{k,k'} e^{i\mathbf{k}\cdot\mathbf{r}} \left(l_{\mathrm{ex}}^2 k^2 + N_{k,yy} \right) a_{k'} a^*_{k'-k} \,.
\end{aligned}
\tag{6}
$$

In the above formulas, we have used the following relations for the magnetostatic field [2, 3]: $\mathbf{h}_\mathrm{m}(\mathbf{m}_0) = -\mathbf{N} \cdot \mathbf{m}_0$, where $\mathbf{N} = \mathrm{diag}\{N_x, N_y, N_z\}$ is the demagnetising tensor, with $N_x + N_y + N_z = 1$, and $\mathbf{m}_0$ is the uniform magnetisation mode ($k = 0$), while for $k \neq 0$, $\mathbf{h}_\mathrm{m}(\mathbf{m}_k) = -\mathbf{N}_k \cdot \mathbf{m}_k$, where $N_{k,ij} = (1 - s_d(k)) k_i k_j / (k^2)$ for $(i,j) \in \{x, y\}$ and $N_{k,zz} = s_d(k)$, with $s_d(k) = (1 - e^{-kd})/(kd)$, and d the thickness of the sample. Notice that for $k \to 0$, $s_d \to 1$, meaning that $N_{k,zz} \to 1$ and $N_{k,ij} \to 0$. For the RF field, we have used the following expansion: $h_{\mathrm{RF}} = \sum_k h_{\mathrm{RF},k} e^{j\mathbf{k}\cdot\mathbf{r}}$. Substituting the Eqs. (3, 5, 6) in Eq. (4), and using Fourier orthogonality, we arrive at the following system of equations that describes the nonlinear dynamics of the

spin-waves amplitudes a_k with $k \neq 0$:

$$
\begin{aligned}
-i\frac{\mathrm{d}a_k}{\mathrm{d}t} =& - h_{\mathrm{RF},k} + \left(H - N_x + l_{\mathrm{ex}}^2 k^2 + \frac{N_{k,yy} + N_{k,zz}}{2}\right) a_k + \frac{N_{k,yy} - N_{k,zz}}{2} a_{-k}^* \\
& - \sum_{k'} \frac{N_{k',xy}}{2} \left(a_{k'} + a_{-k'}^*\right) a_{k-k'} - \frac{N_{k,xy}}{2} \sum_{k'} a_{k'} a_{k'-k}^* \\
& + \frac{1}{2} \sum_{k',k''} h_{\mathrm{RF},k''} a_{k'-k''} a_{k'-k}^* + \frac{N_x}{2} \sum_{k'} |a_{k'}|^2 a_k \\
& - \frac{1}{2} \left(\frac{N_y + N_z}{2} a_0 + \frac{N_y - N_z}{2} a_0^*\right) \sum_{k'} a_{k'} a_{k'-k}^* \\
& + \frac{1}{2} \sum_{k',k''} (l_{\mathrm{ex}}^2 k'^2 + N_{k',xx}) a_{k''} a_{k''-k'}^* a_{k-k'} \\
& - \frac{1}{2} \sum_{k',k''} \left[\left(l_{\mathrm{ex}}^2 k''^2 + \frac{N_{k'',yy} + N_{k'',zz}}{2}\right) a_{k''} + \frac{N_{k'',yy} - N_{k'',zz}}{2} a_{-k''}^*\right] a_{k'-k''} a_{k'-k}^* \\
& + \frac{1}{2} \sum_{k',k'',k} \frac{N_{k'',xy}}{2} a_{k'-k''} a_{k'-k}^* a_{k'''} a_{k'''-k''}^* \,,
\end{aligned}
\tag{7}
$$

and to the following one for the uniform mode $k = 0$:

$$
\begin{aligned}
-i\frac{\mathrm{d}a_0}{\mathrm{d}t} =& - h_{\mathrm{RF},0} + \left(H - N_x + \frac{N_y + N_z}{2}\right) a_0 + \frac{N_y - N_z}{2} a_0^* \\
& - \sum_{k'} \frac{N_{k',xy}}{2} \left(a_{k'} + a_{-k'}^*\right) a_{-k'} \\
& + \frac{1}{2} \sum_{k',k''} h_{\mathrm{RF},k''} a_{k'-k''} a_{k'}^* \\
& - \frac{1}{2} \left[\left(\frac{N_y + N_z}{2} - N_x\right) a_0 + \frac{N_y - N_z}{2} a_0^*\right] \sum_{k'} |a_{k'}|^2 \\
& - \frac{1}{2} \sum_{k',k''} \left[\left(\frac{N_{k'',yy} + N_{k'',zz}}{2} - N_{k',xx}\right) a_{k''} + \frac{N_{k'',yy} - N_{k'',zz}}{2} a_{-k''}^*\right] a_{k'-k''} a_{k'}^* \\
& + \frac{1}{2} \sum_{k',k'',k} \frac{N_{k'',xy}}{2} a_{k'-k''} a_{k'}^* a_{k'''} a_{k'''-k''}^* \,.
\end{aligned}
\tag{8}
$$

All the coefficients of Eq. (7) possess a symmetry with respect to he inversion of k. Then, if exist, the steady states of a_k and a_{-k} will be the same, or if $a_k(0) = a_{-k}(0)$, then $a_k(t) = a_{-k}(t)$ for $t \geq 0$.

The use of the SWM for numerical computations does not give any advantage with respect to standard micromagnetics. The numerical complexity grows with $\mathcal{O}\left(N \log N\right)$ due to the presence of convolutions – similar to micromagnetics solvers [4], where N is the number of discretisation cells of the k-space for the SWM and of the real

space for the micromagnetic code respectively. However, in the next section and in the main text too, we show that, by using general arguments, it is possible to extrapolate from the SWM simplified models that allow us to describe with analytical methods the linear dynamics and the parametric instability of the spin-wave amplitudes.

S2 Linear Dynamics

Let us start considering the linear and conservative dynamics of spin wave amplitudes. In this respect, we take from Eqs. (7) and (8) only the linear terms and do not consider the RF-field term. Then, we have the following equation:

$$-i\frac{\mathrm{d}a_k}{\mathrm{d}t} = A_k a_k + B_k a^*_{-k} \,, \tag{9}$$

where the expressions for A_k and B_k as a function of the material parameters and the k index, for every k, can be directly obtained from the Eqs. (7) and (8). The above equation has a Hamiltonian structure but is not in a diagonal form. In order to preserve the structure and diagonalise it, we perform the following change of variables [2, 5]:

$$\begin{aligned} b_k &= \varepsilon_k a_k + \eta_k a^*_{-k} \,, \\ \varepsilon_k &= \cosh\frac{\psi_k}{2} \,, \\ \eta_k &= \sinh\frac{\psi_k}{2} \,, \\ \tanh\psi_k &= \frac{B_k}{A_k} \,. \end{aligned} \tag{10}$$

According to this transformation, Eq. (9) takes the following form:

$$-i\frac{\mathrm{d}b_k}{\mathrm{d}t} = \varepsilon_k\left(-i\frac{\mathrm{d}a_k}{\mathrm{d}t}\right) - \eta_k\left(i\frac{\mathrm{d}a^*_{-k}}{\mathrm{d}t}\right) = \omega_k b_k \,, \tag{11}$$

where the relations:

$$\omega_k = \sqrt{A_k^2 - B_k^2} = \begin{cases} \sqrt{\left(H - N_x + l_{\mathrm{ex}}^2 k^2 + N_{k,yy}\right)\left(H - N_x + l_{\mathrm{ex}}^2 k^2 + N_{k,zz}\right)} \,, \\ \sqrt{\left(H - N_x + N_y\right)\left(H - N_x + N_z\right)} \,, \end{cases} \tag{12}$$

are in the order from top to bottom, the dispersion relation for $k \neq 0$ and Kittel's frequency $k = 0$. The variables b_k will be called spin-wave normal modes because their conservative dynamic is governed by the equations of a set of independent harmonic oscillators.

The inclusion of damping effects in the model follows the same procedure used to derive terms due to the conservative torque of the Landau-Lifshitz equation. In this respect, one can easily show that the linear dynamics, including damping effects and

the RF-field term, is described by the following equation:

$$-i\frac{\mathrm{d}a_k}{\mathrm{d}t} = -h_{\mathrm{RF},k} + (1+i\alpha)(A_k a_k + B_k a^*_{-k})\,. \tag{13}$$

Then, if we rewrite it in terms of the spin-wave normal modes, we get the following equation:

$$-i\frac{\mathrm{d}b_k}{\mathrm{d}t} = -h_k + \omega_k b_k + i\alpha(A_k b_k + B_k b^*_{-k})\,, \tag{14}$$

where $h_k = \varepsilon_k h_{\mathrm{RF},k} - \eta_k h^*_{\mathrm{RF},-k}$. The amplitude of the RF-field is a sinusoidal function in time, therefore, it can be decomposed into two circular polarised fields: one with clockwise rotation in the complex plane h_k^+ and the other with counter-clockwise rotation h_k^-. This means that each b_k has both components (b_k^+, b_k^-) too. We are interested in the spin-wave normal mode amplitudes close to the resonance condition $\omega_k \approx \omega_{\mathrm{RF}}$. Then, with good approximation, the relation $b_k \approx b_k^+$ can be assumed. When Eq. (14) is written in a rotating complex plane with angular frequency ω_{RF} and only slow varying terms are kept, by using the phasor notation $b_k(t) = \bar{b}_k \exp(i\omega_{\mathrm{RF}}t)$, we have the following relation:

$$\bar{b}_k = \frac{-\bar{h}_k^+}{(\omega_{\mathrm{RF}} - \omega_k) - i\alpha A_k}\,. \tag{15}$$

The symmetry $a_k(t) = a_{-k}(t)$ discussed in the previous section implies the same symmetry on the b_k independently of the dynamics (as a special case, the same property holds for Eq. (15)). This is used in the main text for the derivation of the threshold RF field for the spin-wave parametric instability.

S3 Spin-Wave Parametric Instability

At low excitation power, the spin-wave dynamics remain linear, and only Damon-Eshbach (DE) spin waves and the uniform mode are excited. Their amplitudes can be calculated using the linearised equations of motion, involving the spin-wave amplitudes.

In the following, we assume that the RF frequency is such that the uniform mode is off-resonance, so it is not significantly excited. Independent of the RF frequency, MMM images reveal that beyond a certain RF power, additional spin waves are excited, despite not coupling directly to the RF field. These additional modes exhibit wave vectors in directions different from that of the RF excitation, which is a hallmark of parametric instability.

To explain this, two types of nonlinear magnon scattering processes are considered:

1. *Three-magnon scattering process*: The SWM (see Eq. (7) and Eq. (8)) involves a summation of terms, each of the following form: $h_{\mathrm{RF},k''} a_{k'-k''} a^*_{k'-k}$. The relevant contributions are those where a parametrically excited mode couples with both an RF-driven DE spin wave mode and the RF field. This restricts the summation to terms with $k' = 0$ or $k' = k'' + k$.

2. *Four-magnon scattering process:* This process involves two DE spin waves and two parametrically excited spin waves with opposite wave vectors ($\pm k$). The relevant terms are of the form: $a_{k'-k''}a_{k''}a^*_{k'-k}$ (see Eq. (7)). Restricting again to $k'=0$ or $k'=k''+k$, we identify interactions where DE spin waves $a_{\pm k''}$ scatter into parametrically, not directly RF-field-excited modes $a_{\pm k}$. This leads to the following simplified SWM:

$$
\begin{aligned}
-i\frac{\mathrm{d}a_k}{\mathrm{d}t} = &(1+i\alpha)\left(A_k a_k + B_k a^*_{-k}\right) + \frac{1}{2}\sum_{k'\in\{k_{\mathrm{DE}}\}} h_{\mathrm{RF},k'}\left(a^*_{k'}a_k + a_{-k'}a^*_{-k}\right)\\
&-\frac{1}{2}\sum_{k'\in\{k_{\mathrm{DE}}\}}\left(C_{k'}\left|a_{k'}\right|^2 + B_{k'}a^*_{k'}a^*_{-k'}\right)a_k\\
&-\frac{1}{2}\sum_{k'\in\{k_{\mathrm{DE}}\}}\left|a_{k'}\right|^2\left(C_k a_k + B_k a^*_{-k}\right)\,,
\end{aligned}
\tag{16}
$$

where

$$
\begin{aligned}
A_k &= H - N_x + l^2_{\mathrm{ex}}k^2 + (N_{k,yy}+N_{k,zz})/2\,,\\
B_k &= (N_{k,yy}-N_{k,zz})/2\,,\\
C_k &= l^2_{\mathrm{ex}}k^2 + (N_{k,yy}+N_{k,zz})/2\,.
\end{aligned}
$$

Here, the coefficients A_k, B_k, C_k describe dipolar and exchange contributions, and the set $\{k_{\mathrm{DE}}\}$ includes the Damon-Eshbach (DE) spin wave modes.

S3.1 Parametric Equations

In the following, the derivation of the parametric terms that appear in the equations of spin-wave normal modes is given. The starting point is Eq. (16). Each term on the right-hand side transforms according to Eq. (11). Let us consider the nonlinear terms due to the RF-field. The following transformation relation results:

$$
\begin{aligned}
h_{\mathrm{RF},k'}(a^*_{k'}a_k + a_{-k'}a^*_{-k}) \to &\left(\varepsilon_k h_{\mathrm{RF},k'} - \eta_k h^*_{\mathrm{RF},-k'}\right)\left(a^*_{k'}a_k + a_{-k'}a^*_{-k}\right)\\
\approx &\frac{1}{\omega_k\omega_{k'}}\left[\left(A_kA_{k'} + B_kB_{k'} + \omega_k\omega'_k\right)h^+_{k'}\right.\\
&\left. - \left(A_kB_{k'} + B_kA_{k'}\right)h^{-*}_{-k'}\right]b_{-k'}b^*_{-k}\,,
\end{aligned}
\tag{17}
$$

where the approximated equality symbol means that only parametric terms have been considered. The last equation represents the first parametric term that augments the linear model of spin-wave normal mode dynamics and will allow us to investigate the threshold of parametric instability. The nonlinear term describing the four-magnon scattering process in the Eq. (16), from which the second parametric term is derived,

transforms according to the following relation:

$$
\begin{aligned}
&|a_{k'}|^2 \left[(C_k + C_{k'})\, a_k + B_k a^*_{-k}\right] + B_{k'} a^*_{k'} a^*_{-k'} a_k \\
&\to \quad |a_{k'}|^2 \left[(C_k + C_{k'})\left(\varepsilon_k a_k - \eta_k a^*_{-k}\right) + B_k \left(\varepsilon_k a^*_{-k} - \eta_k a_k\right)\right] \\
&\qquad + B_{k'} (a^*_{k'} a^*_{-k'} \varepsilon_k a_k - a_{-k'} a_{k'} \eta_k a^*_{-k}) \\
&\approx \quad \frac{B_k B_{k'}}{2\omega_k \omega_{k'}} \left[(C_k + C_{k'} - A_k)\, b^2_{k'} - A_{k'} b_{k'} b_{-k'}\right] b^*_{-k} \,.
\end{aligned}
\tag{18}
$$

When both parametric terms are inserted into the equation of dynamics for $b_k(t)$, and $b_{k'}$ is expressed according to Eq. (15), one obtains the following equation:

$$
\begin{aligned}
-i\frac{\mathrm{d}b_k}{\mathrm{d}t} =& (\omega_k + i\alpha A_k) b_k \\
&+ \sum_{k' \in \{k_{\mathrm{DE}}\}} \left(\frac{\left(\chi^+_{k,k'} h^+_{k'} + \chi^-_{k,k'} h^{-*}_{k'}\right) h^+_{k'}}{\omega_{\mathrm{RF}} - \omega_{k'} - i\alpha A_{k'}} + \frac{\zeta_{k,k'} h^{+2}_{k'}}{(\omega_{\mathrm{RF}} - \omega_{k'} - i\alpha A_{k'})^2} \right) b^*_{-k} \,,
\end{aligned}
\tag{19}
$$

where:

$$
\begin{aligned}
\chi^+_{k,k'} &= \frac{1}{2\omega_k \omega_{k'}} \left(A_k A_{k'} + B_k B_{k'} + \omega_k \omega'_k\right) \,, \\
\chi^-_{k,k'} &= -\frac{1}{2\omega_k \omega_{k'}} \left(A_k B_{k'} + B_k A_{k'}\right) \,, \\
\zeta_{k,k'} &= -\frac{B_k B_{k'}}{2\omega_k \omega_{k'}} \left(H - N_x\right) \,.
\end{aligned}
\tag{20}
$$

The linear resonance condition of the DE modes for $\omega_{k'} \approx \omega_{\mathrm{RF}}$ maximises the magnitude of the coefficient of the parametric terms and then minimises the threshold for the parametric instability [2]. Moreover, in such a condition, these two types of coefficient scale differently with respect to α. For the yttrium iron garnet (YIG) medium, the value of the damping constant $\alpha \approx 10^{-4}$ is such that the coefficient of the parametric term due to the four-magnon scattering process for $\omega_{k'} \approx \omega_{\mathrm{RF}}$ is several orders of magnitude larger than the coefficient of the three-magnon scattering mediated by the RF field. Therefore, this last one can be safely neglected.

S3.2 Critical Field

Before the instability sets in, we assume DE spin waves oscillate in a steady linear state (see Eq. (15)). Transforming Eq. (16) into the normal-mode basis b_k , and focusing on four-magnon parametric terms, we arrive at:

$$
-i\frac{\mathrm{d}b_k}{\mathrm{d}t} = (\omega_k + i\alpha A_k)\, b_k + \sum_{k' \in \{k_{\mathrm{DE}}\}} \frac{\zeta_{k,k'} h^{+2}_{k'}}{\left(\omega_{\mathrm{RF}} - \omega_{k'} - i\alpha A_{k'}\right)^2} b^*_{-k} \,. \tag{21}
$$

This equation represents a parametric oscillator, where the second term triggers instability by coupling b_k to the conjugate mode b^*_{-k}. Following Suhl's approach [2], the

critical RF field for instability is:

$$h_{\mathrm{RF,crit}} = \left(\frac{(\omega_{\mathrm{RF}} - \omega_k)^2 + \alpha^2 A_k^2}{\left| \sum_{k' \in \{k_{\mathrm{DE}}\}} \frac{\zeta_{k,k'} \hat{h}_{k'}^{+2}}{(\omega_{\mathrm{RF}} - \omega_{k'} - i\alpha A_{k'})^2} \right|^2} \right)^{\frac{1}{4}}, \tag{22}$$

where we have used the following relation: $\overline{h}_{k'}^{+} = h_{\mathrm{RF}}\, \hat{h}_{k'}^{+}$, with h_{RF} the amplitude of the RF field.

S3.3 Inclusion of the Uniform Mode Dynamics

When the RF-field frequency approaches the uniform (Kittel) mode, its contribution can no longer be ignored. Following Suhl [2], additional parametric terms of the type $b_0^2 b_{-k}^*$ must be added to Eq. (21). Such terms are obtained via the transformation terms of the SWM given by the product of one among a_0^2, a_0^{*2}, $|a_0|^2$ with one of the terms a_k, a_{-k}^*. Interestingly, Eqs. (21) and (22) still hold but $k' \in \{0, k_{\mathrm{DE}}\}$, where

$$\zeta_{k,0} = B_0 \frac{A_k (B_0 + B_k) + B_k (A_0 - \omega_0) - B_k D_k}{4\omega_0 \omega_k} - \frac{(A_0 + \omega_0) A_k E_k}{4\omega_0 \omega_k} \tag{23}$$

with

$$\begin{aligned} A_0 &= H - N_x + (N_y + N_z)/2 \\ B_0 &= (N_y - N_z)/2 \\ D_k &= (N_y + N_z)/2 - N_x + (N_{k,yy} + N_{k,zz})/2 - N_{k,xx} \\ E_k &= (N_y + N_z)/2 - l_{\mathrm{ex}}^2 k^2 - N_{k,xx}\ . \end{aligned}$$

In Fig. 2c and in Fig. S1, we show the threshold field distribution in k-space for two RF frequencies according to the theory discussed in the Methods section of the main text. Given the RF frequency, the threshold field values are significantly lower on the dispersion curve in the k-space corresponding to $\omega_k = \omega_{\mathrm{RF}}$, and, as expected, the higher the efficiency of the coplanar waveguide (CPW), the lower the critical field value.

S3.4 Connection to Suhl's Instability

In materials like YIG with low damping $\alpha \approx 10^{-4}$, only wave vectors k' with $\omega_{k'} \approx \omega_{\mathrm{RF}}$, such that the CPW coupling efficiency is not zero, contribute significantly to the denominator in Eq. (22). We define $\tilde{k}$ the k-space indices such that this relation is satisfied. Then, the sum over the interval $\{0, k_{\mathrm{DE}}\}$ can be replaced in good approximation by the sum over $\{\tilde{k}\}$. In this respect, when the CPW efficiency peaks at $\{\tilde{k}\}$,

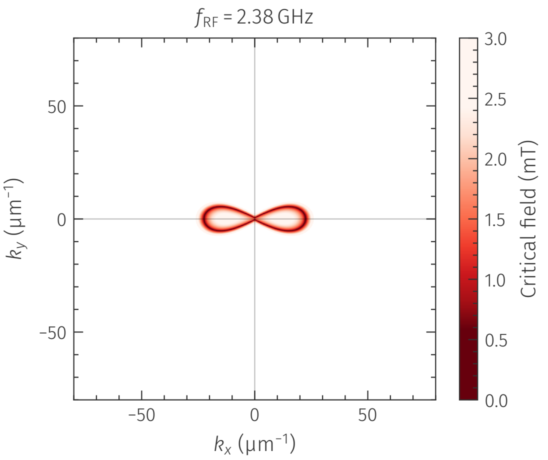

Supplemental Fig. S1: Theoretical threshold field distribution in k-space for a frequency of $f_{\mathrm{RF}} = 2.38\,\mathrm{GHz}$

Eq. (22) becomes:

$$h_{\mathrm{RF,crit}} \approx \frac{\left[\left(\omega_{\mathrm{RF}} - \omega_k\right)^2 + \alpha^2 A_k^2\right]^{\frac{1}{4}} \left[\left(\omega_{\mathrm{RF}} - \omega_{\tilde{k}}\right)^2 + \alpha^2 A_{\tilde{k}}^2\right]^{\frac{1}{2}}}{\left(2\left|\zeta_{k,\tilde{k}} \hat{h}_{\tilde{k}}^{+2}\right|\right)^{\frac{1}{2}}} \,. \tag{24}$$

This equation reduces to Suhl's expression for the second-order instability [2] for $\tilde{k} = 0$, and for the CPW coupling efficiency peaked at $k = 0$. Therefore, the spin-wave instability described here represents a generalised form of Suhl's second-order instability in which the modes responsible for the parametric excitation are, in principle, all those that directly couple to the RF field due to the coupling efficiency of the CPW. In our case, these modes are a subset of the DE spin waves.

S3.5 Threshold Field for Parametric Excitation of Spin Waves

The threshold field for spin wave parametric excitation corresponds to the minimum in the k-space of the critical field expressed by Eq. (24). The minimization of the critical field can be done in two steps. The first one corresponds to set the resonance condition $\omega_k = \omega_{\tilde{k}} = \omega_{\mathrm{RF}}$ in the above relation that can be written as:

$$h_{\mathrm{RF,crit}} \approx \sqrt{\frac{A_k A_{\tilde{k}}}{\left|B_k B_{\tilde{k}}\right|}} \, \frac{\alpha^{3/2} \omega_{\mathrm{RF}} \sqrt{A_{\tilde{k}}}}{\left|\hat{h}_{\tilde{k}}^{+}\right| \sqrt{H - N_x}} \,. \tag{25}$$

The second step corresponds to select among the infinite spin wave modes that satisfy the resonance condition, those that minimize the factor $A_k/|B_k|$. This factor takes into

account the elliptical polarization of the spin wave amplitudes a_k and in the limiting case where it becomes circular, $B_k \to 0 \Rightarrow h_{\mathrm{RF,crit}} \to \infty$. Then, the occurrence of the spin wave parametric instability is forbidden when the magnetic equilibrium is uniform and out of the film plane (z-direction). The minimum of the factor $A_k/|B_k|$ depends on the RF field frequency. For frequency values considered in the experiments, we found that it is minimum when $k = \tilde{k}$, which corresponds to the DE spin-wave modes such that $\omega_{\tilde{k}} = \omega_{\mathrm{RF}}$. In this respect, Eq. (25) becomes

$$h_{\mathrm{RF,thr}} = \min_k h_{\mathrm{RF,crit}} \approx \frac{A_{\tilde{k}}}{|B_{\tilde{k}}|} \frac{\alpha^{3/2}\omega_{\mathrm{RF}}\sqrt{A_{\tilde{k}}}}{\left|\hat{h}^+_{\tilde{k}}\right|\sqrt{H - N_x}} . \tag{26}$$

When the resonance condition is not satisfied anymore, the critical field increases rapidly, namely, the parametric excitation occurs only for spin-wave modes in a narrow region in the k-space, where the resonance condition $\omega_k \approx \omega_{\tilde{k}} \approx \omega_{\mathrm{RF}}$ is satisfied.

S3.6 Magnetization Deflection-Angle at the Instability Condition

The excitation of certain spin wave modes produces a deflection-angle between the local magnetization vector and the direction of the magnetic equilibrium at each point of the film. Once the magnetization component perpendicular to the equilibrium direction ($m_\perp$) is known, such angle can be determined by the following relation:

$$\theta(\mathbf{r}, t) = \arctan\left(\frac{m_\perp}{\sqrt{1 - m_\perp^2}}\right)\Bigg|_{m_\perp \ll 1} \approx m_\perp , \tag{27}$$

where according to Eqs. (3) and (10), one has

$$m_\perp = |m_\pm| = \sqrt{\sum_{k,k'} (\varepsilon_{k'} b_{k'} - \eta_{k'} b^*_{-k'})(\varepsilon_{k''} b^*_{-k''} - \eta_{k''} b_{k''}) e^{j(\mathbf{k}' + \mathbf{k}'')\cdot \mathbf{r}}} . \tag{28}$$

Prior to the spin-wave parametric instability, the only spin-wave modes excited are those $\tilde{k} \in \{k_{\mathrm{DE}}\} : \omega(\tilde{k}) = \omega_{\mathrm{RF}}$, namely, spin waves resonant with the RF field. The spin wave dynamics before the instability is assumed linear, and the steady oscillations are described by Eq. (15). By considering it and the following property of the spin-wave normal-mode amplitudes: $b_k(t) = b_{-k}(t)$, Eq. (28) reduces to

$$m_\perp = \sqrt{\left[\left(\varepsilon^2_{\tilde{k}} + \eta^2_{\tilde{k}}\right)\left|b_{\tilde{k}}\right|^2 - \varepsilon_{\tilde{k}}\eta_{\tilde{k}}\left(b^2_{\tilde{k}} + b^{*2}_{\tilde{k}}\right)\right]}\left|\cos \tilde{k} y\right| . \tag{29}$$

The maximum of the deflection angle corresponds to the maximum of the perpendicular component of the magnetization field. Then, if the above relation is maximized

with respect to the point and time instant, one can arrive to the following relation:

$$m_\perp = \frac{h_{\mathrm{RF}}|\hat{h}^+_{\tilde{k}}|}{\alpha\sqrt{A_{\tilde{k}}\omega_{\mathrm{RF}}}}\,. \tag{30}$$

At the instability condition, the RF field value is given by Eq. (25), and therefore the perpendicular magnetization component can be expressed as:

$$m_{\perp,\max} \approx \frac{A_{\tilde{k}}}{|B_{\tilde{k}}|}\sqrt{\frac{\alpha\,\omega_{\mathrm{RF}}}{H - N_x}}\,. \tag{31}$$

In the case $f_{\mathrm{RF}} = 2.38\,\mathrm{GHz}$, $m_{\perp,\max} \approx 0.03$ that corresponds to a maximum magnetization deflection-angle of $\theta_{\max} \approx 2^\circ$, while for $f_{\mathrm{RF}} = 9.00\,\mathrm{GHz}$, $m_{\perp,\max} \approx 0.15$ and $\theta_{\max} \approx 10^\circ$. Such values are consistent with the assumption of linearity that has been used for the derivation of the threshold RF field value. Indeed, it corresponds to small tilting angles of the local magnetization with respect to the equilibrium direction.

References

[1] Mayergoyz, I.D., Bertotti, G., Serpico, C.: Nonlinear Magnetization Dynamics in Nanosystems. Elsevier Series in Electromagnetism. Elsevier Science, Amsterdam (2009). https://doi.org/10.1016/b978-0-08-044316-4.x0001-1

[2] Suhl, H.: The theory of ferromagnetic resonance at high signal powers. Journal of Physics and Chemistry of Solids **1**(4), 209–227 (1957) https://doi.org/10.1016/0022-3697(57)90010-0

[3] Guslienko, K.Y., Slavin, A.N.: Magnetostatic green's functions for the description of spin waves in finite rectangular magnetic dots and stripes. Journal of Magnetism and Magnetic Materials **323**(18–19), 2418–2424 (2011) https://doi.org/10.1016/j.jmmm.2011.05.020

[4] Magnetization Geometrical Integration Code - Ma.G.I.Co. http://wpage.unina.it/mdaquino/index_file/MaGICo.html Accessed 2025-07-25

[5] Zakharov, V.E., L'vov, V.S., Starobinets, S.S.: Spin-wave turbulence beyond the parametric excitation threshold. Soviet Physics Uspekhi **17**(896) (1975) https://doi.org/10.1070/PU1975v017n06ABEH004404
