# Supplementary material for: Soft-X-ray momentum microscopy of nonlinear magnon interactions
Source: Nat Phys. 2026 Jun 5;22(7):1160–5. doi: 10.1038/s41567-026-03318-z (PMC13423880; doi:10.1038/s41567-026-03318-z)
Supplement: Supplementary file 2 — Peer Review File [file 41567_2026_3318_MOESM2_ESM.pdf]

nature portfolio

Peer Review File

Soft-X-ray momentum microscopy of nonlinear magnon interactions below 100-nm wavelength

Corresponding Author: Dr Steffen Wittrock

Version 0:

Reviewer comments:

Reviewer #1

(Remarks to the Author)
The authors present magnon momentum microscopy (MMM), a soft X-ray scattering technique that directly images magnons in two-dimensional momentum space with high sensitivity and photon efficiency. Using this method, they access sub-100-nm exchange-dominated magnons in yttrium iron garnet films and uncover rich nonlinear interactions, including four-magnon scattering processes that redistribute spin-wave populations omnidirectionally. The authors demonstrate that MMM enables rapid mapping of spin-wave dispersions and reveals higher harmonics and parametric instabilities, and thus can potentially serve as a universal method to access coherent spin-wave excitations with frequencies up to THz and with element specificity.

The manuscript is well written, timely, and with great potential. It is of course a demonstration for ferromagnetic YIG films which are already studied to a great extent, however for a benchmark study this is indeed a good choice. I do, however, have a few major and minor questions, which I want the authors to address prior to publication.

Major questions:

1. What is the physical reason for using such a square-shaped mask and why does the beamstop look like a cross?
2. The authors report a four-magnon parametric instability in YIG films. While they provide the RF power at which it occurs, could they estimate the spin-deflection angle at which it occurs? I find this very important if coherent excitations are discussed.
3. I am also wondering what is the origin of the SW excitations which form a circular shape. For the case of a 1D grating it seems rather unlikely to have concentric ring excitations to which a circular Fourier pattern would correspond.
4. I am wondering why in Fig. 2f excitation along the f_rf maximizes at q_perp = 0 while other orders acquire a finite q_perp.
5. Why in Fig. 3 the spin-wave dispersions at the multiple f_rf (2rf, 3rf..) seem to have lower frequencies compared to the original? It looks rather counterintuitive.
6. The authors discuss application of the technique to THz waves, obviously referring to AFM systems. Can the authors discuss applicability of their technique to collinear AFM or non-collinear AFM? Would it require higher sensitivity or would one have to switch from XMCD to XMLD, which obviously has orders of magnitude smaller yield?

Minor questions:

1. Please indicate a coplanar waveguide and the grating coupler in Fig. 1b. It also would be nice to show Fig. 1b in some other place within Fig. 1 otherwise it does not appear in alphabetical order.
2. I would refrain from saying that the developed model goes beyond LLG which is linear. Generally LLG is nonlinear; it is the linearized version that is linear.

Overall, this is a strong and exciting experimental advance. Addressing the major points above (especially the physical origin of the ring, the quantitative estimate of coherent amplitude, and the AFM/XMLD applicability) will significantly increase the manuscript’s impact and clarity.

Reviewer #2

(Remarks to the Author)
General report
==============

The authors describe a new X-ray based experimental technique to determine directly a 2D map of spin waves of a given frequency in a thin film, which they call Magnon Momentum Microscopy (MMM). It is based on the scattering of X-rays on linear fronts of spin waves, initially excited directionally using a co-planar wave guide and grating couplers, later converted to all directions above a given power threshold via magnon non-linear interactions. The technique is clearly novel and provides quick and unlimited access to the 2D map, which can be a very powerful method of thin-film material characterization of spin waves. This alone warrants publication in Nat. Phys. The authors also provide a detailed analysis and modelling of the four-magnon scattering process underlying in their case the non-linear physics required for the technique. This is valuable both from the physical point of view, but also technically to support the analysis of data from the measurements. The manuscript is written with great care and is clear, so are the figures.

I support publication in Nat. Phys. after necessary revision. Detailed comments are provided in the following, some more or less focused, which I advise the authors to consider. Most importantly, I consider that the authors should better explain the generality/limits of the technique, some aspects remaining completely undiscussed. This cannot remain as such while highlighting a new technique: a discussion of applicability is required. A first aspect is the potential application to materials with damping larger than YIH. Does the decay of magnon intensity away from the strip line changes the measurements, their analysis, the occurrence or efficiency of the four-magnon scattering process? A second aspect is the cut-off of high-frequency / short wavelengths (both should be discussed as they are linked to different instrumental aspects). While I understand that measurement is possible, I would like to hear more about the generation side. Are there limitations expected arising from the CPW/grating? Is there an impact of strip-line and coupler thickness or side roughness, which would scatter waves already from their emission? Finally, the authors claim repeatedly that they address (and the technique is specifically suitable to address) the exchange regime and 100-nm barrier. The exchange regime depends on the type of SW, material, thin thickness etc. This claim should be discussed, again contributing to highlight the generality of the claim.

Finally, a personal comment, I leave this also in the hand of the Editor to discuss: I find the term “Microscopy” misleading. The name is in general linked to real-space observation. I find that a name such as “Mapping” would be much more suitable, and the authors can keep the shortcut MMM.

Detailed comments
=================
• General
o Vectors are sometimes indicated with arrows (may text), then with boldface (Supplementary information). Please rationalize.
o Subscripts: sometimes correctly used with slanted for variables ate upright for names, such as in M_s. this is the case in the main text. However, is done differently in the Supplementary, s is slanted in M_s.
o The reading of subscripts combining two different meanings is sometimes difficult, e.g., h_effz (see Eq;2 from Supplementary). First, again z could be slanted. Second, a comma may be used to separate the two subscripts. (check other situations in the entire manuscripts)
o The usual rule of embedded brackets may be used everywhere for better readability, eg the sentences below Eq.(6) in Supplementary: {…[…(…)…]…} [note: it is suitably used in some places, such as Eq.(8)]
o
• Abstract
o For a non-expert, at this stage the link between exchange interactions and non-linearities will be obscure -> Could you please re-arrange?
o Mention of two-dimensional: at this stage, may not be clear whether this comes from considering a thin-film (which comes at the end of the sentence), or a specific output of the techniques
• Introduction
o “are considered a promising vehicle to overcome the limitations of predominant CMOS technology in low-power analogue and digital computing as well as signal processing”. Please reformulate. There are deep and shared doubts in device players about the broad use of magnonics, owing to the weak efficiency of excitation and detection (voltage-magnon-voltage) conversion. This does not remove any of the excellent achievement in physics in the paper, but being more realistic in the claims for application would be fair.
o In the detection schemes, please add the recent demonstration of detection with magnetoresistance by Bailleul and collaborators, which is very promising.
o “Light, however, can be used as a more versatile and non-invasive probe of magnons”
  Very short and two arguments, if you wish to keep, please elaborate
  Versatile: should be explained why the previous techniques are less”
  Non-invasive: should be explained how the previous techniques are invasive.
o You jump directly from BLS to the required use of X-rays. Could you elaborate on other techniques? There have been nice demonstrations of magnon probing with NV center microscopy or TEM techniques. How they compare should be described to the reader
o “Across the entire dispersion plane remains a critical challenge” The link with possible limitations of RIXS and STXM have not been made explicit -> Please discuss. Please also explain at this stage why sensitivity matters and why the two techniques mentioned fail.
o Note: some aspects are covered in the discussion part, but at the stage of the introduction this sounds like an un supported claim.
• Magnon Momentum Microscopy
o “Across the entire dispersion plane remains a critical challenge”: two things differ between STXM and MMM: the scheme of measurement, and the total flux of X-rays. Please disentangle both, to better understand the underlying mechanism: de we

gain mostly because there are more photons here than with a Fresnel lens in STXM?
• FIG.2
o Second row: the frequency may be mentioned with a label, similar to the first row, to ease the reading of the figure for all frames, without having to read the entire caption.
o Comment for the caption or related text: so far you have described wavelength (eg for the limitation of existing techniques), now you switch to k vectors. Could you translate these (eg those reported in FIG.2) to wavelength to facilitate the reading by the reader? This conversion is provided in the discussion, which I find too late.
• High-Resolution Mapping of the Spin-Wave Dispersion
o Traditional methods typically fail to capture the magnonic behaviour in this regime. Please explain in more detail, for the methods mentioned in the intro (plus possibly NV center, TEM). Is it the deep sub-micrometer wavelength that you claim? Or the 2D?
• Techniques
o Conversion of dB into OE field in the YIG would be helpful (it is discussed later on in the SWM, because indeed it is the relevant parameter for the threshold of non-linearity).

Reviewer #3

(Remarks to the Author)
In the manuscript by Wittrock et al., the authors probed the dispersion and nonlinear interaction of magnons by means of quasi-elastic, resonant magnetic soft-X-ray scattering. They reported the excitation of nonlinear magnons by off-resonant excitation frequency, among which the wavelength of magnons can be as short as 100~nm. I find the experimental tools are advanced and impressive. The reported results are interesting and valuable to the community of magnetization dynamics. However, the physics mechanism appears not sufficiently new since the theoretical model is standard, and a careful comparison between the theory and experiment is not performed. So I find it difficult to recommend the publication of the manuscript of the present form in Nature Physics regarding several main concerns:

(1)The authors addressed ``This magnon momentum microscopy (MMM) can access magnons directly in momentum space with remarkable sensitivity and high photon efficiency up to THz frequencies and down to few-nanometre wavelengths." But in the main text, the excitation frequency is about 10~GHz, and the wavelength of the excited magnon is about 100~nm. So, although the resolution of magnon momentum microscopy could be very sharp, as the authors envision, it is not reported in the present manuscript. In other words, if this resolution can be appropriately reported in this manuscript, it would attract much attention from the magnon community, such that the publication of the manuscript is warranted.

(2)The theoretical model about the nonlinear interaction, including the three-magnon and four-magnon processes, is proper. But there is no careful comparison with the experiment. Although the formula is presented, can the authors use the formula to reproduce the excitation of nonlinear magnons and compare with the experiment?

(3)In the theoretical model, can the authors compare the efficiency of three-magnon and four-magnon processes? Which one is dominant under different excitation powers?

(4)It appears that the theoretical model cannot explain key features of the observation since the authors addressed on page 9 “A complete theoretical framework to explain these harmonics is still lacking and remains a subject for future study.” I suggest that the authors compare the theoretical model and observation to clearly state the status of the understanding, i.e., which part can be explained and which part is still a mystery.

Version 1:

Reviewer comments:

Reviewer #1

(Remarks to the Author)
The authors have addressed all my questions and concerns convincingly. In my opinion, the modified manuscript is robust and rigorous and has reached the standards of Nature Physics. I can now recommend this paper for a publication in its current form.

Reviewer #2

(Remarks to the Author)
The authors have suitably addressed my first three main comments: limits of the technique in terms of material; cut-off frequencies; length-scale barrier versus exchange-dominated spin waves.

I keep finding the term “Microscopy” misleading, even though it is used by some. It does not fit the ordinary vocabulary, referring to small objects (I checked in the Merriam Webster and on Wikipedia). Microscopy bears the concept of looking at things too small to be seen by the naked eye. By definition, the eye des not see the reciprocal space. There is no magnification involved in MMM.

The authors have also addressed my minor points. Therefore, I am happy to recommend publication in Nat. Phys., although I remain reluctant with the concept of microscopy in the reciprocal space.

Reviewer #3

(Remarks to the Author)
I thank the authors for the detailed reply. However, my mind is not changed, and I believe that this manuscript is not suitable for publication in Nature Physics.

The main limitations of this manuscript are twofold.

On one hand, the technique's potential "THz frequencies and down to few-nanometre wavelengths" is not demonstrated at all by the authors. This renders the technique of magnon momentum microscopy not go beyond microwave spectroscopy and Brillouin light scattering.

On the other hand, the updated comparison between the theoretical model and experimental measurements does not reveal sufficiently new physics. The instability in the parametric pumping is well established, and the related nonlinear dynamics have been well studied in the literature for decades. So the instability for magnons with finite wave vectors is expected.

Open Access This Peer Review File is licensed under a Creative Commons Attribution 4.0 International License, which permits use, sharing, adaptation, distribution and reproduction in any medium or format, as long as you give appropriate credit to the original author(s) and the source, provide a link to the Creative Commons license, and indicate if changes were made.
In cases where reviewers are anonymous, credit should be given to 'Anonymous Referee' and the source.
The images or other third party material in this Peer Review File are included in the article's Creative Commons license, unless indicated otherwise in a credit line to the material. If material is not included in the article's Creative Commons license and your intended use is not permitted by statutory regulation or exceeds the permitted use, you will need to obtain permission directly from the copyright holder.
To view a copy of this license, visit https://creativecommons.org/licenses/by/4.0/

Answers to the reviewers

First, we would like to thank all three referees for their reports, the time they spent on our manuscript, and the points they have raised. In the following, we address all their comments in order to improve our manuscript. In addition to the revised manuscript, we also provide a colour-marked version highlighting all major changes. In this letter, we mark the

reviewer comments in black

and our

response in blue with *quotations from the manuscript in italic font style.*

All line-numbers refer to the colour-marked manuscript.

General Correction to the Manuscript:

Only after submission of the manuscript, we noticed an error in the sample description and that we have actually used a sister sample of the one presented in Ref. 12 [Baumgaertl, K. et al., *Nano Letters* 20(10), 7281–7286 (2020)] with the main difference of a grating-coupler (GC) period of 400 nm instead of 200 nm.

To be very clear and transparent on this matter, this difference does not affect any of the general capabilities of our magnon momentum microscopy (MMM), nor the experimental or theoretical findings related to the nonlinear magnon-magnon interactions described in the manuscript.

We have amended the antenna parameters in the manuscript text and methods accordingly. In addition, we also updated Extended Data Fig. 2 displaying the calculated spectrum of excitation efficiencies of our antenna. Importantly, the excitation spectrum for the 400-nm GC is very similar to that for 200-nm GC, with GC modes occurring at the same wave vectors but at higher orders. We sincerely apologise for this mistake.

Reviewer Comments:

Reviewer #1

The authors present magnon momentum microscopy (MMM), a soft X-ray scattering technique that directly images magnons in two-dimensional momentum space with high sensitivity and photon efficiency. Using this method, they access sub-100-nm exchange-dominated magnons in yttrium iron garnet films and uncover rich nonlinear interactions, including four-magnon scattering processes that redistribute spin-wave populations omnidirectionally. The authors demonstrate that MMM enables rapid mapping of spin-wave dispersions and reveals higher harmonics and parametric instabilities, and thus can potentially serve as a universal method to access coherent spin-wave excitations with frequencies up to THz and with element specificity.

The manuscript is well written, timely, and with great potential. It is of course a demonstration for ferromagnetic YIG films which are already studied to a great extent, however for a benchmark study this is indeed a good choice. I do, however, have a few major and minor questions, which I want the authors to address prior to publication.

We thank the reviewer for the positive assessment and in the following thoroughly address all the comments in order to further improve the manuscript.

Major questions:

1. What is the physical reason for using such a square-shaped mask and why does the beamstop look like a cross?

In the previous version of our manuscript, the purpose of the mask was not described with sufficient clarity. We have now revised the text in the Methods section (L346ff):

"To restrict the region probed by the X-ray beam, we used a square-shaped mask that defines the illuminated area on the sample. Its main purpose is to confine the illumination to a topographically uniform region, thereby suppressing unwanted topographic (charge) scattering from surrounding structures such as the CPW or GC, which would otherwise obscure the magnetic scattering from the spin waves. The mask was fabricated from a Cr/Au multilayer (total thickness ≈ 1 µm) deposited on a SiN membrane, into which a 16 × 16 µm² square aperture was milled using a Ga^+ focused-ion beam (FIB). It was then transferred onto the front side of the sample and attached by FIB-assisted Pt deposition. The aperture defines the illuminated detection region in the bare YIG, with its centre located approximately 11 µm away from the CPW. The SiN support electrically insulates the metallic mask from the CPW, preventing any shorting.

Introducing such an aperture inevitably produces additional scattering from the mask edges. To minimize this effect, the square-shaped mask was designed such that the edge-induced scattering occurs primarily along two perpendicular directions defined by the mask edges. These scattering streaks are effectively blocked by a cross-shaped beamstop placed in the detection path. For this reason, the mask was intentionally rotated by 45° with respect to the expected Damon–Eshbach (DE) magnon wave-vector direction $q_\parallel$. This geometry ensures that the regions affected by mask-edge scattering coincide with the beamstop coverage and are thus blanked out in the MMM images, while the magnetic scattering signal remains unaffected. In future implementations of the method, the mask may be substituted by a well-focused and well-defined X-ray beam properly positioned on the sample."

2. The authors report a four-magnon parametric instability in YIG films. While they provide the RF power at which it occurs, could they estimate the spin-deflection angle at which it occurs? I find this very important if coherent excitations are discussed.

We thank the reviewer for highlighting this aspect. In the Supplemental Information, we also detail the theoretical calculus and add a new section entitled "Magnetization Deflection-Angle at the Instability Condition". In this section, we derive an analytical expression for the magnetization deflection angle at the threshold RF field corresponding to the onset of parametric spin-wave excitation. The estimated values of this deflection angle are now included in the subsection "Experimental Implications" in the Methods section. There, we further added a discussion on the validity of the linear approximation used for the Damon–Eshbach (DE) spin-wave dynamics below the instability threshold. In particular, we show that the growth of the magnetization deflection angle is limited by the onset of the parametric instability, ensuring that the assumption of linearity remains accurate for RF fields below the threshold.

In the main text, we now add a comment (L174ff):

"*Furthermore, the magnetization deflection angle at the parametric-instability threshold remains small (θ_{max} ≈ 2° at f_{RF} = 2.38 GHz and θ_{max} ≈ 10° at f_{RF} = 9.00 GHz; see Supplementary Information).*"

And in the Method section we add (L492ff):

"*In the Supplemental Information, we present calculations of the maximum magnetization deflection angle θ_{max} at the threshold RF field for parametric excitation of spin waves. For f_{RF} = 2.38 GHz, we find θ_{max} ≈ 2°, and for f_{RF} = 9.00 GHz, θ_{max} ≈*

10°. These results indicate that the growth of the DE spin-wave amplitude is self-limited by the onset of parametric instability. The small values of θ_{max} further confirm the consistency of the linear approximation used to describe the DE spin-wave dynamics below the threshold."

3. I am also wondering what is the origin of the SW excitations which form a circular shape. For the case of a 1D grating it seems rather unlikely to have concentric ring excitations to which a circular Fourier pattern would correspond.

   The referee is correct that, at first sight, a circular diffraction pattern is unexpected, since a 1D grating primarily excites spin waves along a single wave-vector direction $q_\parallel$. Indeed, at low excitation power, we observe two clear diffraction peaks only along the $q_\parallel$ direction. However, above a certain power threshold, the diffraction pattern becomes circular, already pointing at a nonlinear origin, cf. Fig 2e,f.

   We can further neglect direct excitation of magnon-modes with a sizeable $q_\perp$ component by the GC and CPW, e.g. due to edge roughness, cf. our response to issue #2 of reviewer 2 and Fig. R2 for more details. Note that also the detection of directly excited BWV modes by the GC or CPW can be ruled out, as they cannot enter the X-ray field of view in the MMM experiment, see Fig. 1a and Extended Data Fig. E4.

   As we further show, this isotropic occupation of magnon modes arises from a nonlinear instability process, predominantly driven by four-magnon scattering, which redistributes the initially excited Damon–Eshbach mode into secondary modes in arbitrary directions.

   In the manuscript, we discuss this as follows:
   L149ff: "*[...] the two anticipated diffraction peaks in the $q_\parallel$ direction are accompanied by a distinct elliptical scattering ring (Fig. 2a). This ring clearly indicates the population of magnon modes across all directions of propagation. [...]*"

   We furthermore improve our description in L156ff:
   "Our analysis reveals that the mechanism responsible for populating magnon modes across all directions is a spin-wave parametric resonance driven by four-magnon scattering. In this process, two directly excited DE modes couple to two secondary magnons of arbitrary wave vector $\pm k_{SW}$. The coupling strength is proportional to the population of the DE modes. Parametric excitation becomes possible only when the population of the DE modes reaches a threshold value corresponding to a threshold radio frequency (RF) field (or power) amplitude. The critical field for the parametric excitation–threshold field distribution in momentum space can be derived from the SWM (see Supplementary Information) and is presented in Fig. 2c for an excitation frequency of f_{RF} = 9.00 GHz."

4. I am wondering why in Fig. 2f excitation along the f_rf maximizes at q_perp = 0 while other orders acquire a finite q_perp.

   At the fundamental frequency, f_{RF}, the excitation maximum occurs at $q_\perp = 0$ corresponding to the most efficient coupling perpendicular to the stripe geometry and exclusively along $q_\parallel$. For higher harmonic or fractional-order excitations, the maxima appear at finite $q_\perp$, reflecting more complex redistribution of magnon modes. This intriguing feature highlights the rich information content provided by MMM. While understanding these deep nonlinear interactions in full detail is beyond the scope of the present work, our focus here is to demonstrate the general capabilities of the MMM technique. Currently, we are investigating how these amplitude distributions

relate to the underlying nonlinear processes, and a concise interpretation of the fractional parametric modes is not yet established.

We add to the discussion of Fig. 2f in the manuscript (L204ff):
"*The appearance of these modes highlights the deeply nonlinear character of the strongly driven system. While a detailed theoretical understanding of these fractional harmonics is beyond the scope of this study, they underscore the rich information content accessible with MMM and remain a topic for future investigation.*"

5. Why in Fig. 3 the spin-wave dispersions at the multiple f_rf (2rf, 3rf..) seem to have lower frequencies compared to the original? It looks rather counterintuitive.

   This apparent lower frequency arises from the nature of the measurement: MMM directly detects the magnon wave vector rather than its frequency. The higher-order harmonic signals are therefore observed at larger q values. While the excitation frequency of the external drive remains fixed at f_{RF}, the nonlinear system generates harmonic modes at integer multiples of this frequency, which are accompanied by correspondingly larger wave vectors that are detected.

   We agree that this may appear counterintuitive from the perspective of frequency-based detection techniques, and have added a clarification (L227ff):
   "*Note that the higher-harmonic branches appear at lower frequencies than the fundamental because the dispersion in Fig. 3a is shown as a function of the excitation frequency f_{RF}. The actual magnon frequencies, however, obey $f_{SW} = n f_{RF}$, and MMM measures the corresponding wave vectors.*"

6. The authors discuss application of the technique to THz waves, obviously referring to AFM systems. Can the authors discuss applicability of their technique to collinear AFM or non-collinear AFM? Would it require higher sensitivity or would one have to switch from XMCD to XMLD, which obviously has orders of magnitude smaller yield?

   We thank the reviewer for addressing this important issue about probing AFM magnons in the THz range. In principle, even for a collinear AFM, there can be non-zero net-magnetization, $\mathbf{M}(t) \neq 0$, in the case of dynamically excited coherent AFM spin waves [Blank et al., *Phys. Rev. Lett.* **131**, 096701 (2023), new Ref. 39]. Depending on the geometry, i.e. magnon-propagation vector $\mathbf{k}_{sw}$ with respect to the direction of the dynamic magnetization $\mathbf{M}(t)$, XMCD can still be a feasible contrast mechanism. In case there is no projection of the dynamic magnetization vector $\mathbf{M}(t)$ onto the X-ray wave vector $\mathbf{k}_i$ possible, XMLD might serve as an alternative contrast mechanism. In both cases, the *magnetic contrast* will be significantly lower than for FM material systems. However, due to the high photon efficiency of the MMM technique, we are confident that our method is sensitive enough to access AFM magnons in future experiments.

   We have extended the sentence about probing AFM magnons in the "Perspective" section (L321ff) and have added Ref. 39 to the manuscript:
   "*It can be extended to detect magnons up to THz frequencies [37, 38] in antiferromagnetic materials by accessing a magnon-driven dynamic net magnetization either by XMCD [39], or depending on the geometry by exploiting X-ray magnetic linear dichroism (XMLD) as a contrast mechanism.*"

Minor questions:

1. Please indicate a coplanar waveguide and the grating coupler in Fig. 1b. It also would be nice to show Fig. 1b in some other place within Fig. 1 otherwise it does not appear in alphabetical order.

   We have now indicated the coplanar waveguide (CPW) and grating coupler (GC) in Panel **b** of Fig. 1. We cannot find a better placement of Panel **b** without significantly increasing the size of the whole figure, or by completely rearranging the sketch in Panel **b**. Accordingly, we would like to keep the arrangement of Panel **b** and order of labels as is.

   [redacted]

   Figure R1: Updated version of Fig. 1, including indicators and labels for the coplanar waveguide (CPW) and grating coupler (GC).

2. I would refrain from saying that the developed model goes beyond LLG which is linear. Generally LLG is nonlinear; it is the linearized version that is linear.

   The referee is correct. We have revised the sentence (L303ff) to:
   "*To interpret this behaviour, we developed a spin-wave model that reformulates the classical Landau–Lifshitz description in terms of spin-wave amplitudes, enabling a clear analysis of the nonlinear interaction terms.*"

Overall, this is a strong and exciting experimental advance. Addressing the major points above (especially the physical origin of the ring, the quantitative estimate of coherent amplitude, and the AFM/XMLD applicability) will significantly increase the manuscript's impact and clarity.

We thank the reviewer for the positive note and hope that we could address all major and minor points above.

Reviewer #2

<u>General report</u>

The authors describe a new X-ray based experimental technique to determine directly a 2D map of spin waves of a given frequency in a thin film, which they call Magnon Momentum Microscopy (MMM). It is based on the scattering of X-rays on linear fronts of spin waves, initially excited directionally using a co-planar wave guide and grating couplers, later converted to all directions above a given power threshold via magnon non-linear interactions. The technique is clearly novel and provides quick and unlimited access to the

2D map, which can be a very powerful method of thin-film material characterization of spin waves. This alone warrants publication in Nat. Phys. The authors also provide a detailed analysis and modelling of the four-magnon scattering process underlying in their case the non-linear physics required for the technique. This is valuable both from the physical point of view, but also technically to support the analysis of data from the measurements. The manuscript is written with great care and is clear, so are the figures.

We thank the reviewer for pointing out the two main aspects of the work and the positive evaluation of it.

1. I support publication in Nat. Phys. after necessary revision. Detailed comments are provided in the following, some more or less focused, which I advise the authors to consider. Most importantly, I consider that the authors should better explain the generality/limits of the technique, some aspects remaining completely undiscussed. This cannot remain as such while highlighting a new technique: a discussion of applicability is required. A first aspect is the potential application to materials with damping larger than YIG. Does the decay of magnon intensity away from the strip line changes the measurements, their analysis, the occurrence or efficiency of the four-magnon scattering process?

   We agree with the reviewer that a discussion of the applicability of MMM towards other materials than YIG is interesting and relevant. While the (experimental) **analysis** of MMM is generally sample independent, a key distinction must be made between **(i) occurrence or efficiency of the four-magnon scattering process** and the intrinsic **(ii) measurement** capabilities of the MMM technique.

   Regarding (i): The onset or efficiency of nonlinear interactions such as four-magnon scattering, as well as the decay of magnon intensity, are governed by the magnetic properties of the investigated material—such as its damping, anisotropy, and spin-wave dispersion. Stimulated by the reviewer, we discuss the relevance of these parameters for the nonlinear instability process in the Supplementary Information and in the section “Experimental Implications” of the Methods section now in more detail.

   Regarding (ii): The decreased propagation length for materials exhibiting larger damping than YIG, will inevitably reduce the spin-wave amplitude as well as the accessible interaction volume of propagating spin waves with any probe, e.g. photons, electrons, or antenna structures. For the case of MMM, the illuminated sample area can be readily reduced down to diameters of about 1 μm using apertures while maintaining the overall sensitivity and photon flux at state-of-the-art synchrotron radiation facilities. Using dedicated soft-X-ray optics, focus diameters even below 100 nm can be achieved, however implying an increase in beam divergence and hence resulting in a broadening of the diffraction signal in reciprocal space. In analogy to a transmission grating, the diffraction from only a few spin-wave periods also reduces and broadens the diffraction signal in reciprocal space, which can decrease the efficiency of the MMM technique but does not pose a general limitation for the study of nonlinear propagation effects of spin waves.

   Following the reviewer’s remark, we have added the following statement into our “Perspective” (L318ff):
   “*MMM can readily be adapted towards material systems with shorter spin-wave propagation lengths than YIG by reducing the soft-X-ray-magnon interaction volume using smaller X-ray apertures or dedicated focussing schemes down to the sub-μm regime.*”

2. A second aspect is the cut-off of high-frequency / short wavelengths (both should be discussed as they are linked to different instrumental aspects). While I understand that measurement is possible, I would like to hear more about the generation side. Are there limitations expected arising from the CPW/grating? Is there an impact of strip-line and coupler thickness or side roughness, which would scatter waves already from their emission?

   We emphasize that the MMM **detection** principle is entirely independent of the **generation** scheme. In this first demonstration, a coplanar waveguide (CPW) with a grating coupler (GC) is used as a well-established and controllable source of spin waves, but it is not an essential component of the method and can readily be replaced by other excitation mechanisms, such as optical or THz-driven sources.

   Regarding the **generation** side in our experiment, the CPW and GC provide microwave magnetic fields, which generate oscillating torques on the spins in the underlying YIG film (for detailed information see our Refs. [10,12]). In the linear regime, only the CPW/GC eigenmodes are excited, see Extended Data Fig. 2a. For a given excitation frequency, the excitation strength depends on the matching of wave vectors of the allowed eigenmode in YIG with the CPW/GC modes.

   Edge roughness can, in principle, give rise to weak additional excitation channels by broadening the excitation maxima and introducing small wave-vector components along the long axis of the GC stripes and the CPW. However, the excitation efficiency associated with such statistically distributed inhomogeneities is expected to be negligible compared to that of the deliberately engineered CPW/GC modes. However, to quantify potentially generated wavevectors, we performed a Fourier analysis of the CPW edge roughness, shown in Fig. R2. The resulting wave-vector components are confined to small magnitudes, well below those of interest in the present experiment, and lie entirely within the region blocked by the beamstop. Consequently, any spin waves potentially generated by edge roughness neither contribute significantly to the excitation process nor appear in the detected MMM signal.

   The frequency bandwidth of the CPW depends on its design and dielectric cladding. Supported frequencies could go up to the 100 GHz frequency regime for an appropriately designed CPW. Depending on the generator and integrated cables, we limited our experiments to ≈ 10 GHz.

[redacted]

Figure R2: Supported wave vectors by the edge roughness. Apart from the fact that a major effect of the edges is not expected in terms of generated RF magnetic field and hence

magnon excitation, note that the supported wave vectors are small and, hence i) off-resonant for the measurements we show, and ii) would be covered behind the beamstop.

Considering the **detection** limits, MMM itself imposes no intrinsic cut-off at high frequencies, and in terms of wave vector is limited only by the photon wavelength (≈ 1.75 nm in the soft X-ray regime) on the upper side, while the lower limit is defined by the beamstop geometry.

In the "Discussion" section, we address this as follows (L257ff):
"*The upper limit for accessible wave vectors in MMM is ultimately set by the overall diffraction limit, specifically, by the wavelength of the scattered soft-X-ray photons, $\lambda_{ph} \approx 1.75$ nm, corresponding to $2k_{ph} \approx 7200\,\mu m^{-1}$. In our current setup, the maximum detectable wave vector is further limited by the detector geometry, while the beamstop obscures features at $q < 20\,\mu m^{-1}$. However, these setup parameters are readily adaptable to specific experimental requirements.*"

Furthermore, we specify in the Methods section (L382ff):
"*The relative distances between the sample, beamstop, and CCD X-ray detector define the accessible wave vectors in the experiment. Here, we choose the sample-to-CCD distance to z_0 = 260 mm, which defines the scattering signal position on the CCD chip through $d = k_{sw} z_0 / k_i$, with k_{sw} the wave number of the magnon, $k_i = 2\pi\, E_{ph}/(hc)$ the wave number of the incident soft X-rays. Adjusting these parameters and the beamstop position allows further flexibility regarding the accessibility of magnon wave vectors.*"

3. Finally, the authors claim repeatedly that they address (and the technique is specifically suitable to address) the exchange regime and 100-nm barrier. The exchange regime depends on the type of SW, material, film thickness etc. This claim should be discussed, again contributing to highlight the generality of the claim.

   We thank the reviewer for pointing out this inaccuracy in our manuscript. Indeed, the 100-nm barrier is not **generally** applicable as distinction of the exchange regime. Commonly, for the exchange length of YIG, l_{ex} = 16 nm, the magnon regime for $\lambda_{SW} < 2\pi\, l_{ex} \approx 100$ nm is referred to as *exchange-dominated* in literature. And indeed for the **specific case** of the DE and BWV-modes in our 100-nm-thin YIG film, we observe a clear parabolic shape of the dispersion relation in Fig. 3a, which we assign to this *exchange-dominated* regime.

   In order to be clear on this classification, we substitute the phrase "exchange-dominated" when falsely used as a synonym for "sub-100-nm" in our manuscript. Moreover, we changed the relevant part in the introduction as follows (L45ff):
   "*As the magnon wavelength decreases, short-range exchange interactions begin to dominate. In the prototypical magnonic material yttrium iron garnet (YIG), this crossover typically occurs for wavelengths below 100 nm. A major challenge in accessing this largely unexplored regime is the ability to reliably excite and detect such short-wavelength modes [5].*"

4. Finally, a personal comment, I leave this also in the hand of the Editor to discuss: I find the term "Microscopy" misleading. The name is in general linked to real-space observation. I find that a name such as "Mapping" would be much more suitable, and the authors can keep the shortcut MMM.

   We are aware that the term microscopy is often associated with real-space imaging.

However, the terminology "momentum microscopy" is already well established in other fields—most notably in photoelectron and electron momentum microscopy (see, e.g., Tusche et al., *Ultramicroscopy* 159, 520-529 (2015))—where it similarly refers to direct reciprocal-space imaging with high momentum resolution.

In this context, the term microscopy appropriately reflects the two-dimensional imaging nature and high-resolution mapping capability of our technique, albeit in momentum rather than real space. The alternative term mapping, while descriptive, could be ambiguous as it does not clearly convey the full imaging character and precision of the method. We therefore believe that Magnon Momentum Microscopy (MMM) most accurately captures both the conceptual and methodological essence of our approach.

Detailed comments

General

5. Vectors are sometimes indicated with arrows (main text), then with boldface (Supplementary information). Please rationalize.
6. Subscripts: sometimes correctly used with slanted for variables and upright for names, such as in M_s. This is the case in the main text. However, is done differently in the Supplementary, s is slanted in M_s.
7. The reading of subscripts combining two different meanings is sometimes difficult, e.g., h_effz (see Eq;2 from Supplementary). First, again z could be slanted. Second, a comma may be used to separate the two subscripts. (check other situations in the entire manuscripts)
8. The usual rule of embedded brackets may be used everywhere for better readability, eg the sentences below Eq.(6) in Supplementary: {…[…(…)…]…} [note: it is suitably used in some places, such as Eq.(8)]

   We have carefully addressed these remarks and standardized the typesetting throughout the manuscript and Supplementary Information. Vector notation, subscripts, and embedded brackets have been made consistent according to the suggested conventions to improve clarity and readability.

Abstract

9. For a non-expert, at this stage the link between exchange interactions and non-linearities will be obscure -> Could you please re-arrange?
10. Mention of two-dimensional: at this stage, may not be clear whether this comes from considering a thin-film (which comes at the end of the sentence), or a specific output of the techniques

    We thank the reviewer for these valuable comments on the clarity of our abstract. We have rewritten the abstract as follows:
    "*Magnons are quantised collective excitations of long-range ordered spins. At nanometre wavelengths, exchange interactions increasingly govern their dynamics, giving rise to a largely unexplored regime of couplings between magnons and other quasiparticles. Yet, detecting such short-wavelength spin waves has remained a key experimental challenge. Here, we introduce magnon momentum microscopy (MMM)–a quasi-elastic, resonant magnetic soft-X-ray scattering technique that directly images magnon populations across two-dimensional momentum space. Owing to its remarkable sensitivity, MMM can capture nonlinear magnon-magnon interactions over large regions of the dispersion plane. Applying MMM to the prototypical magnonic materials yttrium iron garnet (YIG), we uncover a rich variety*

of previously unobserved nonlinear magnon interactions. With its element specificity, bulk sensitivity, as well as intrinsic access to nanometre-scale wavelengths without frequency limitation, soft-X-ray MMM establishes a powerful and versatile platform for exploring short-wavelength and nonlinear magnonics."

Introduction

11. "are considered a promising vehicle to overcome the limitations of predominant CMOS technology in low-power analogue and digital computing as well as signal processing". Please reformulate. There are deep and shared doubts in device players about the broad use of magnonics, owing to the weak efficiency of excitation and detection (voltage-magnon-voltage) conversion. This does not remove any of the excellent achievement in physics in the paper, but being more realistic in the claims for application would be fair.

    Following the reviewer's suggestion, we have reformulated our "Introduction" (L41ff): "*Spin waves and their quasi-particles, magnons, provide a versatile platform for exploring alternative concepts for wave-based information processing beyond conventional CMOS technology [1]. In particular, nonlinear magnonics has emerged as a promising domain for realizing computing schemes that exploit the intrinsic nonlinearity of magnon interactions [2–4].*"

12. In the detection schemes, please add the recent demonstration of detection with magnetoresistance by Bailleul and collaborators, which is very promising.

    We have now incorporated this promising recent detection approach based on magnetoresistance, as recently demonstrated by Bailleul and collaborators [Rossi et al., *Sci. Adv.* 11, 33 (2025), new Ref. 17], into the introduction.

13. "Light, however, can be used as a more versatile and non-invasive probe of magnons"
    a. Very short and two arguments, if you wish to keep, please elaborate
    b. Versatile: should be explained why the previous techniques are less"
    c. Non-invasive: should be explained how the previous techniques are invasive.

    We agree with the reviewer, that the *versatility* statement was not well explained. The non-invasive character of light-scattering typically relates to the fact that samples must not be altered, e.g. by growing antenna structures on their surface. In order to focus on the main line of argumentation, we have changed the sentence as follows (L60ff):
    "*Light, however, can be used as a more versatile probe of magnons.* Unlike electronic detection, o*ptical methods do not rely on patterned antennas, contacts, or Hall structures, allowing flexible scattering and spectroscopy geometries under largely unconstrained sample conditions.*"

14. You jump directly from BLS to the required use of X-rays. Could you elaborate on other techniques? There have been nice demonstrations of magnon probing with NV center microscopy or TEM techniques. How they compare should be described to the reader
15. "Across the entire dispersion plane remains a critical challenge" The link with possible limitations of RIXS and STXM have not been made explicit -> Please discuss. Please also explain at this stage why sensitivity matters and why the two techniques mentioned fail.
16. Note: some aspects are covered in the discussion part, but at the stage of the introduction this sounds like an unsupported claim.

We very much acknowledge the reviewer's careful thoughts about our introductory text. However, we do not aim to provide a complete *review* of available short-wavelength magnon detection techniques in the limited space of the "Introduction", and hence we have concentrated on light-based techniques here. However, for completeness, we have added the so far missing information on NV centre and TEM techniques to the "Discussion" part of our manuscript, also introducing three new references [Bertelli et al., *Sci. Adv.* **6**, eabd3556 (2020), Zhou et al., *PNAS* 118, (2021), new Ref. 33 & 34] and [Kepaptsoglou et al., *Nature* 644, (2025), new Ref. 35]:

L279ff: "*Magnon probes other than light are often susceptible to variations in the sample environment or rely on stringent external conditions. For example, the resonance frequency of nitrogen-vacancy (NV) centres shifts in external magnetic fields [33,34], complicating measurements. Likewise, the impressive progress achieved by combining scanning transmission electron microscopy (STEM) and electron energy loss spectroscopy (EELS) to access antiferromagnetic magnons across the full Brillouin zone with meV energy resolution [35] relies on strong magnetic electron lenses, which are difficult to reconcile with most ferromagnetic spin-wave materials.*"

Deliberately, we have chosen not to discuss other experimental techniques in the "Introduction". Instead, we do so in the "Discussion" section, where a direct comparison to our MMM results is more meaningful, and we can further explicitly address the role of sensitivity, see also our response to the next issue. To that end, we intentionally kept the description of the limitations of techniques such as RIXS and STXM rather general in the "Introduction". However, the presence of a *detection gap,* that has been so far inaccessible by these existing X-ray techniques, is well-supported by the immediate observation of previously unseen nonlinear magnon interactions with our MMM technique.

Magnon Momentum Microscopy

17. "Across the entire dispersion plane remains a critical challenge": two things differ between STXM and MMM: the scheme of measurement, and the total flux of X-rays. Please disentangle both, to better understand the underlying mechanism: do we gain mostly because there are more photons here than with a Fresnel lens in STXM?

    The improved sensitivity and coverage of MMM compared to STXM originates primarily from the different measurement principle, rather than simply from a higher photon flux. In MMM, the detection occurs directly in reciprocal space via quasi-elastic scattering, where the spin-wave-induced magnetic modulation acts as an effective diffraction grating. This geometry enables the simultaneous detection of all accessible magnon wave vectors within the illuminated area, independent of their real-space location or phase.
    In contrast, STXM relies on real-space imaging, requiring spatial scanning and Fourier transformation to access momentum-space information. This inherently limits sensitivity, acquisition speed and, regarding the need for a focussed beam and synchronized operation, also spatial and temporal resolution.

    In the manuscript, we address this discussion with:
    L269ff: "*STXM has recently demonstrated access to magnons with wavelengths near 100 nm [9, 12]. While powerful in resolving real-space spin dynamics, the sensitivity of STXM to short (and fast) spin-waves is inherently limited by its spatial and temporal resolution, which is dictated by the X-ray optics and pulse duration,*

respectively.”

L287ff: *“What sets MMM apart from other approaches is its combination of high sensitivity, rapid acquisition, and direct 2D access to momentum space without frequency limitations–all within a relatively simple experimental setting. These capabilities originate from the underlying scattering process, where spin-wave-induced magnetic modulations form an effective absorption grating. [...] Operating directly in reciprocal space, MMM bypasses the limitations of inefficient X-ray imaging or dispersion optics, with 2D diffraction patterns that directly encode magnon wave vectors and intensities, eliminating the need for complex reconstruction or modelling.”*

FIG.2

18. Second row: the frequency may be mentioned with a label, similar to the first row, to ease the reading of the figure for all frames, without having to read the entire caption.
19. Comment for the caption or related text: so far you have described wavelength (eg for the limitation of existing techniques), now you switch to k vectors. Could you translate these (eg those reported in FIG.2) to wavelength to facilitate the reading by the reader? This conversion is provided in the discussion, which I find too late.

We changed the figure labels according to the suggestion of the reviewer. In the revised manuscript, we now provide the conversion between wave vector and wavelength earlier in the section “Magnon Momentum Microscopy” (L107ff):
“*For spin waves with a well-defined wave vector $\mathbf{k}_{SW}$ (with wavelength $\lambda_{SW}=2\pi/|\mathbf{k}_{SW}|$), magnetic diffraction peaks of +1st and −1st order emerge in the scattering pattern at [....]*”

[redacted]

Figure R3: Updated Fig. 2, with following changes: Added both parameter labels on every graph, power and frequency. The parameter, which stays the same, is depicted in a lighter gray colour. Also, we change the label for the colour scale of the theory graph from “Threshold field (mT)” to “Critical field (mT)” in order to be more explicit on the mechanism.

High-Resolution Mapping of the Spin-Wave Dispersion

20. Traditional methods typically fail to capture the magnonic behaviour in this regime. Please explain in more detail, for the methods mentioned in the intro (plus possibly NV center, TEM). Is it the deep sub-micrometer wavelength that you claim? Or the 2D?

    We discuss the applicability of other *traditional* methods in capturing nanometre-scale magnons in the “Discussion” section (L263ff). Starting in L287ff, we then highlight the specific advantages of MMM. While today, different techniques can access sub-micrometer wavelength magnons, the specific advantages of MMM are the unique combination of direct access to two-dimensional reciprocal space without frequency limitation, and superb signal-to-noise ratio. This together renders MMM as a very powerful and explorative technique, especially for nonlinear magnonics.

Techniques

21. Conversion of dB into OE field in the YIG would be helpful (it is discussed later on in the SWM, because indeed it is the relevant parameter for the threshold of non-linearity).

    We agree with the reviewer that the conversion from RF excitation power (in dBm) to actual Oersted field strength (in mT) provides an important and quantitative link between experiment and theory. To that end, we have performed COMSOL simulations of the RF field distribution below the coplanar waveguide, see Fig. R4b. We observe that the experimentally generated RF field is consistent with the theoretical threshold field calculations.

[redacted]

Figure R4: Updated Extended Data Fig. E2 including the following changes: a Rescaling to absolute efficiency and correction of sample geometry. **b** New plot of magnetic excitation field distribution below the CPW and GC structure. Red areas depict the CPW and grey areas the GC structure.

In particular, the experimental magnetic excitation field for the experimental RF power of -5.0 dBm that is mainly represented in the manuscript (apart from Fig. 2d-f) is around 1 mT. Comparing this value with the theoretically calculated threshold field

(Fig. 2c and Fig. S1) demonstrates that the parametric instability preferentially emerges in regions where the CPW/GC structure provides the strongest RF magnetic field, i.e. where the excitation efficiency is maximal (Fig. R4a).

We can conclude the following:

- At the higher frequencies around 9 GHz (Fig. 2c), the excitation field overcomes the threshold field only in a very narrow band of frequencies and wave vectors (compare Extended Data Figs. E2 and E3). This is reflected by the well-separated diffraction peaks at frequencies not exactly lying on the resonance (see, e.g., Fig. 1), where we are still below the threshold and clearly in the linear regime. Once being exactly on the resonance (Fig. 2a) however, we overcome the threshold field and observe the occurrence of the instability, i.e. the occurrence of the diffraction ring.
- At low frequencies (Fig. S1), the threshold field is well overcome and hence, the nonlinear instability process is triggered consistently in a broad range of frequencies. This is exactly what we observe experimentally.

The "Experimental Implications" subsection in the Method section contains the following paragraph (L479ff):
"*An order-of-magnitude analysis shows that when the CPW and GC efficiency, cf. Extended Data Fig. E2a,* $|\hat{h}^{+}_{k'}| \approx 5\times10^{-3}$*, the threshold field is* $H_{RF,thr} \approx 1$ *mT, whereas for maximum efficiency (*$\approx 2\times10^{-2}$*), the threshold field decreases to* $H_{RF,thr} \approx 0.01$ *mT. These values are consistent with the experiments: the threshold power value at* f_{RF} *= 9.00 GHz is* $P_{RF} \approx -5.0$ *dBm (see Fig. 2a). This power corresponds, according to the COMSOL calculations presented in the Extended Data Fig. E2b, to an RF field amplitude of 1.3 mT. At lower RF frequencies,* $f_{RF} = 2.38$ *GHz, the same level of RF power excites additional higher-order spin-wave modes of frequencies* $2f_{RF}$*,* $3f_{RF}$ *and* $4f_{RF}$ *(see Fig. 2b). The theoretically estimated threshold field (power) is almost two (one) orders of magnitude smaller than the value at* f_{RF} *= 9.00 GHz. This is due to the higher coupling efficiency of the CPW at lower wave vectors. Therefore, the excitation of higher-order parametric resonances (*$n f_{RF}$*, n = 2, 3, 4) is consistent with the fact that the RF field (power) significantly exceeds the threshold value.*"

Reviewer #3

In the manuscript by Wittrock et al., the authors probed the dispersion and nonlinear interaction of magnons by means of quasi-elastic, resonant magnetic soft-X-ray scattering. They reported the excitation of nonlinear magnons by off-resonant excitation frequency, among which the wavelength of magnons can be as short as 100 nm. I find the experimental tools are advanced and impressive. The reported results are interesting and valuable to the community of magnetization dynamics. However, the physics mechanism appears not sufficiently new since the theoretical model is standard, and a careful comparison between the theory and experiment is not performed.
So I find it difficult to recommend the publication of the manuscript of the present form in Nature Physics regarding several main concerns:

We thank the reviewer for acknowledging our experimental advances and valuing our results on nonlinear magnon interactions. We also agree with the reviewer that in the initial version of the manuscript, the comparison between experiment and theory was not sufficiently detailed. Following also the comments of the other reviewers, we have now substantially improved this part of our manuscript, e.g. within the subsection, "Experimental Implications" in the Methods sections.

However, we respectfully disagree with the judgement that our findings do not represent sufficiently new physics just because the “theoretical model is standard”. Indeed, the foundation of our theoretical model is the LLG equation and our spin-wave model is inspired by the work of Suhl. However, both in experiment and theory we uncover a so far unseen parametric instability for propagating magnons enabled by the unique capabilities of our MMM technique, which provides direct access to short-wavelength, multidirectional nonlinear processes that could not be resolved with existing techniques so far.

1. The authors addressed ``This magnon momentum microscopy (MMM) can access magnons directly in momentum space with remarkable sensitivity and high photon efficiency up to THz frequencies and down to few-nanometre wavelengths.” But in the main text, the excitation frequency is about 10 GHz, and the wavelength of the excited magnon is about 100 nm. So, although the resolution of magnon momentum microscopy could be very sharp, as the authors envision, it is not reported in the present manuscript. In other words, if this resolution can be appropriately reported in this manuscript, it would attract much attention from the magnon community, such that the publication of the manuscript is warranted.

   In the present proof-of-concept demonstration, the **generation** of magnons was indeed limited to wavelengths of about 100 nm in the 10 GHz range. Based on the used microwave CPWs (+ GC), the shortest coherently excited magnon wavelengths can reach the 70–100 nm range–already close to the state-of-the-art limit of spin-wave generation with existing device technology. As discussed in the recent *Magnonics Roadmap* [Flebus et al., *J. Phys.: Condens. Matter* 36, 363501 (2024), Ref. 5], reliable generation of shorter-wavelength magnons remains a major challenge for the field.

   The **detection** limit of our MMM technique is, however, only limited by the X-ray wavelength used for scattering (≈ 1.75 nm), which sets an intrinsic momentum sensitivity up to ≈ 7200 μm^{-1} corresponding to magnons with few-nanometre wavelengths. Regarding frequency, MMM is conceptually broadband because it probes only momentum. The technique is therefore fully compatible with THz-frequencies, e.g. for probing antiferromagnetic magnons, see also our response to Referee 1, Comment #6.

   To that end, we are firmly convinced that our statement “[...] *magnon momentum microscopy (MMM) can access magnons [...] up to THz frequencies and down to few-nanometre wavelengths*” is well justified, although we were not able to experimentally enter this very exciting new regime, yet.

2. The theoretical model about the nonlinear interaction, including the three-magnon and four-magnon processes, is proper. But there is no careful comparison with the experiment. Although the formula is presented, can the authors use the formula to reproduce the excitation of nonlinear magnons and compare with the experiment?

   First, complementing our earlier clarification to the Reviewer, we would like to emphasize that the parametric excitation observed in our experiment exhibits a degree of novelty compared to the standard Suhl-type processes. In our geometry, the CPW and GC directly excites Damon–Eshbach magnons with finite wave vector, and once their population exceeds a critical value, magnons of different wave vectors–but at the same frequency–are parametrically generated. This differs from the conventional scenario where the uniform (k = 0) mode mediates the instability (from Suhl’s seminal work onward, including [Hwang et al., *Sci. Rep. 11*, 14207 (2021)]). The novelty of the theoretical treatment therefore follows directly from the

unconventional parametric pathway revealed by the MMM measurements, even though the mathematical framework of parametric resonance is established.

To address the reviewer's request for a more quantitative comparison, we have substantially expanded both the main text and the Supplementary Information (see also Reviewer 2's question regarding the dBm-to-Oe conversion #21). Specifically, we derive the minimum RF field required for the onset of the parametric instability (the threshold field) and now compare this prediction directly with the experimental conditions. In addition, we include COMSOL simulations of the RF field generated by the CPW at the sample (Fig R4b, new Extended Data Fig. E2b), enabling an accurate estimate of the actual field amplitudes in the experiment. These considerations, together with the COMSOL-based field estimates, are now discussed in a dedicated Methods subsection, "Experimental Implications", where we outline the connection between the model and the experimental conditions.
Using these simulated fields as input, the spin-wave model (SWM) quantitatively reproduces the experimentally observed onset of nonlinear magnons, their wave-vector distribution, and the measured power threshold. This agreement holds while explicitly treating both three-magnon and four-magnon scattering channels (Supplementary Information, Eqs. 7–8). These additions demonstrate that the SWM captures the essential nonlinear dynamics responsible for the multidirectional occupation of modes observed in the MMM images.

3. In the theoretical model, can the authors compare the efficiency of three-magnon and four-magnon processes? Which one is dominant under different excitation powers?

   At all excitation powers relevant to our experiment, the four-magnon process dominates the onset of the observed nonlinear magnon dynamics, which we justify in the Supplementary Information (L678ff) as follows:
   "*The linear resonance condition of the DE modes for $\omega_{k'} \approx \omega_{RF}$ maximises the magnitude of the coefficient of the parametric terms and then minimises the threshold for the parametric instability [30]. Moreover, in such a condition, these two types of coefficient scale differently with respect to α. For the yttrium iron garnet (YIG) medium, the value of the damping constant $\alpha \approx 10^{-4}$ is such that the coefficient of the parametric term due to the four-magnon scattering process for $\omega_{k'} \approx \omega_{RF}$ is several orders of magnitude larger than the coefficient of the three-magnon scattering mediated by the RF field. Therefore, this last one can be safely neglected.*"

4. It appears that the theoretical model cannot explain key features of the observation since the authors addressed on page 9 "A complete theoretical framework to explain these harmonics is still lacking and remains a subject for future study." I suggest that the authors compare the theoretical model and observation to clearly state the status of the understanding, i.e., which part can be explained and which part is still a mystery.

   We thank the reviewer for the comment. The model reproduces the experimentally observed instability threshold, the multidirectional population of magnons, and the corresponding wave-vector distribution with quantitative agreement (see main text and Supplementary Information). In the revised manuscript, we now perform a more detailed quantitative comparison between the model and the measurements (see former reviewer comments). This establishes that the observed rings arise from a four-magnon parametric process, which is fully accounted for in our analysis.
   The present model does not describe all features observed at higher excitation powers. In particular, the appearance of (sub-)harmonics and the detailed azimuthal

redistribution of magnon intensities are not captured within the current framework, as these likely involve additional nonlinear mechanisms beyond the standard three- and four-magnon interactions included here. A complete theoretical description of these phenomena will require further developments and is beyond the scope of the present work, whose primary goal is to introduce MMM as a new experimental technique for momentum-resolved magnon imaging. The main purpose of our theoretical model is to capture the essential nonlinear processes underlying the formation of the elliptical magnon rings and to verify the magnetic origin of the MMM signals.

To make this discussion with the reviewer explicit in the manuscript, we have added the following clarification to the discussion of Fig. 2f (L201ff):
"*As the power increases, a fundamental dispersion ring forms, followed by additional parametric processes that generate spin waves at fractional harmonics of the fundamental frequency (f_{SW} = m/8 f_{RF}, with m = 3 to 14, except for m = 4 and 10). The appearance of these modes highlights the deeply nonlinear character of the strongly driven system. While a detailed theoretical understanding of these fractional harmonics is beyond the scope of this study, they underscore the rich information content accessible with MMM and remain a topic for future investigation.*"

Answers to the reviewers (2nd review)

Reviewer Comments:

Reviewer #1

The authors have addressed all my questions and concerns convincingly. In my opinion, the modified manuscript is robust and rigorous and has reached the standards of Nature Physics. I can now recommend this paper for a publication in its current form.

We thank the reviewer for the very positive assessment and the recommendation.

Reviewer #2

The authors have suitably addressed my first three main comments: limits of the technique in terms of material; cut-off frequencies; length-scale barrier versus exchange-dominated spin waves.
I keep finding the term "Microscopy" misleading, even though it is used by some. It does not fit the ordinary vocabulary, referring to small objects (I checked in the Merriam Webster and on Wikipedia). Microscopy bears the concept of looking at things too small to be seen by the naked eye. By definition, the eye des not see the reciprocal space. There is no magnification involved in MMM.
The authors have also addressed my minor points. Therefore, I am happy to recommend publication in Nat. Phys., although I remain reluctant with the concept of microscopy in the reciprocal space.

We thank the reviewer for the positive assessment of our manuscript and for recommending publication. We also appreciate the reviewer's careful reflection on terminology. However, we remain convinced that "magnon momentum microscopy" most accurately reflects the methodological nature of our technique. The term "momentum microscopy" is already well established in related fields (e.g. photoelectron momentum microscopy, cf *Nat. Mater.* **16**, 615–621 (2017) or *Nat. Phys.* **21**, 1093–1099 (2025)), where it denotes full-field, two-dimensional imaging in reciprocal space with high momentum resolution. Our usage follows this established convention. With our "microscopy" terminology, we want to particularly stress that our technique provides **direct, parallel imaging** of magnon populations in **2D** momentum space with **high resolution**, which is the defining characteristic of microscopy as opposed to point-by-point spectroscopy, as indicated by the term "mapping".

Reviewer #3

I thank the authors for the detailed reply. However, my mind is not changed, and I believe that this manuscript is not suitable for publication in Nature Physics.

The main limitations of this manuscript are twofold.

On one hand, the technique's potential "THz frequencies and down to few-nanometre wavelengths" is not demonstrated at all by the authors. This renders the technique of magnon momentum microscopy not go beyond microwave spectroscopy and Brillouin light scattering.

On the other hand, the updated comparison between the theoretical model and experimental measurements does not reveal sufficiently new physics. The instability in the parametric pumping is well established, and the related nonlinear dynamics have been well studied in the literature for decades. So the instability for magnons with finite wave vectors is expected.

We thank the reviewer for the careful reading of our manuscript and for the continued engagement with our work. While we respect the reviewer's perspective, we would like to clarify our position on the two main concerns.

1. *THz and few-nanometer access:*

   We agree that the present demonstration is performed in the microwave regime (≈ 10 GHz) and accesses wavelengths down to ≈ 70 nm. Our statement regarding THz frequencies and nanometre wavelengths refers to the intrinsic detection capability of MMM, which is fundamentally set by the X-ray wavelength and the accessible scattering geometry, rather than by the present excitation scheme. In particular, while MMM places **no fundamental restriction on frequency** (as it probes momentum rather than oscillation rate), the accessible wave-vector range is determined by the X-ray wavelength and experimental geometry, both of which define a well-known and calculable upper momentum limit. This limit lies deep in the exchange-dominated regime and extends far beyond the wave vectors accessed in the present work.

   We believe that we have carefully described the current experimental constraints and, importantly, they arise from limitations in magnon generation rather than from the detection principle. Establishing MMM as a direct, full-field momentum-resolved detection platform is therefore an essential step toward experimentally accessing precisely the short-wavelength and high-frequency regime highlighted by the reviewer. No established technique offers MMM's unique combination of sensitivity, fidelity, and momentum-space coverage required to access this regime.

2. *Novelty of the nonlinear physics:*

   We fully acknowledge that parametric magnon instabilities have been studied extensively for decades. However, the key novelty of our work lies not in revisiting classical Suhl-type instabilities, but in experimentally resolving – in full momentum space – the multidirectional population of finite-wave-vector magnons driven by a **four-magnon process** from directly excited **Damon–Eshbach modes**. The instability described goes beyond **Suhl's description**, which typically involves the **Kittel mode** as the main component of the nonlinear instability. We are not aware of any study that has previously observed or predicted parametric magnon instabilities that lead to a multidirectional population of magnon modes, as we describe here.

   Furthermore, in conventional experiments, parametric instabilities are typically inferred indirectly (e.g., through bulk signals or selected wave-vector detection). In contrast, MMM enables direct, two-dimensional momentum-resolved imaging of the instability and its wave-vector distribution in a single acquisition. The quantitative agreement between the spin-wave model and the observed instability threshold and

dispersion rings demonstrates that the nonlinear population of finite-k magnons can be directly resolved and imaged experimentally. This provides the first direct visualization of a multidirectional population of parametrically generated magnons emerging from directly excited Damon–Eshbach modes.

Therefore, our work substantially advances the field by (i) demonstrating direct momentum-resolved access to nonlinear magnon dynamics and (ii) the observation of a multidirectional finite-wave-vector instability emerging from directly excited modes.

Answers to the reviewers (1st review)

First, we would like to thank all three referees for their reports, the time they spent on our manuscript, and the points they have raised. In the following, we address all their comments in order to improve our manuscript. In addition to the revised manuscript, we also provide a colour-marked version highlighting all major changes. In this letter, we mark the

reviewer comments in black

and our

response in blue with *quotations from the manuscript in italic font style.*

All line-numbers refer to the colour-marked manuscript.

General Correction to the Manuscript:

Only after submission of the manuscript, we noticed an error in the sample description and that we have actually used a sister sample of the one presented in Ref. 12 [Baumgaertl, K. et al., *Nano Letters* 20(10), 7281–7286 (2020)] with the main difference of a grating-coupler (GC) period of 400 nm instead of 200 nm.

To be very clear and transparent on this matter, this difference does not affect any of the general capabilities of our magnon momentum microscopy (MMM), nor the experimental or theoretical findings related to the nonlinear magnon-magnon interactions described in the manuscript.

We have amended the antenna parameters in the manuscript text and methods accordingly. In addition, we also updated Extended Data Fig. 2 displaying the calculated spectrum of excitation efficiencies of our antenna. Importantly, the excitation spectrum for the 400-nm GC is very similar to that for 200-nm GC, with GC modes occurring at the same wave vectors but at higher orders. We sincerely apologise for this mistake.

Reviewer Comments:

Reviewer #1

The authors present magnon momentum microscopy (MMM), a soft X-ray scattering technique that directly images magnons in two-dimensional momentum space with high sensitivity and photon efficiency. Using this method, they access sub-100-nm exchange-dominated magnons in yttrium iron garnet films and uncover rich nonlinear interactions, including four-magnon scattering processes that redistribute spin-wave populations omnidirectionally. The authors demonstrate that MMM enables rapid mapping of spin-wave dispersions and reveals higher harmonics and parametric instabilities, and thus can potentially serve as a universal method to access coherent spin-wave excitations with frequencies up to THz and with element specificity.

The manuscript is well written, timely, and with great potential. It is of course a demonstration for ferromagnetic YIG films which are already studied to a great extent, however for a benchmark study this is indeed a good choice. I do, however, have a few major and minor questions, which I want the authors to address prior to publication.

We thank the reviewer for the positive assessment and in the following thoroughly address all the comments in order to further improve the manuscript.

Major questions:

1. What is the physical reason for using such a square-shaped mask and why does the beamstop look like a cross?

   In the previous version of our manuscript, the purpose of the mask was not described with sufficient clarity. We have now revised the text in the Methods section (L346ff): *"To restrict the region probed by the X-ray beam, we used a square-shaped mask that defines the illuminated area on the sample. Its main purpose is to confine the illumination to a topographically uniform region, thereby suppressing unwanted topographic (charge) scattering from surrounding structures such as the CPW or GC, which would otherwise obscure the magnetic scattering from the spin waves. The mask was fabricated from a Cr/Au multilayer (total thickness ≈ 1 μm) deposited on a SiN membrane, into which a 16 × 16 μm² square aperture was milled using a Ga^+ focused-ion beam (FIB). It was then transferred onto the front side of the sample and attached by FIB-assisted Pt deposition. The aperture defines the illuminated detection region in the bare YIG, with its centre located approximately 11 μm away from the CPW. The SiN support electrically insulates the metallic mask from the CPW, preventing any shorting.*

   Introducing such an aperture inevitably produces additional scattering from the mask edges. To minimize this effect, the square-shaped mask was designed such that the edge-induced scattering occurs primarily along two perpendicular directions defined by the mask edges. These scattering streaks are effectively blocked by a cross-shaped beamstop placed in the detection path. For this reason, the mask was intentionally rotated by 45° with respect to the expected Damon–Eshbach (DE) magnon wave-vector direction $q_{\parallel}$. This geometry ensures that the regions affected by mask-edge scattering coincide with the beamstop coverage and are thus blanked out in the MMM images, while the magnetic scattering signal remains unaffected. In future implementations of the method, the mask may be substituted by a well-focused and well-defined X-ray beam properly positioned on the sample."

2. The authors report a four-magnon parametric instability in YIG films. While they provide the RF power at which it occurs, could they estimate the spin-deflection angle at which it occurs? I find this very important if coherent excitations are discussed.

   We thank the reviewer for highlighting this aspect. In the Supplemental Information, we also detail the theoretical calculus and add a new section entitled "Magnetization Deflection-Angle at the Instability Condition". In this section, we derive an analytical expression for the magnetization deflection angle at the threshold RF field corresponding to the onset of parametric spin-wave excitation. The estimated values of this deflection angle are now included in the subsection "Experimental Implications" in the Methods section. There, we further added a discussion on the validity of the linear approximation used for the Damon–Eshbach (DE) spin-wave dynamics below the instability threshold. In particular, we show that the growth of the magnetization deflection angle is limited by the onset of the parametric instability, ensuring that the assumption of linearity remains accurate for RF fields below the threshold.

   In the main text, we now add a comment (L174ff):
   "*Furthermore, the magnetization deflection angle at the parametric-instability threshold remains small (θ_{max} ≈ 2° at f_{RF} = 2.38 GHz and θ_{max} ≈ 10° at f_{RF} = 9.00 GHz; see Supplementary Information).*"

   And in the Method section we add (L492ff):

"In the Supplemental Information, we present calculations of the maximum magnetization deflection angle θ_{max} at the threshold RF field for parametric excitation of spin waves. For f_{RF} = 2.38 GHz, we find $\theta_{max} \approx 2°$, and for f_{RF} = 9.00 GHz, $\theta_{max} \approx 10°$. These results indicate that the growth of the DE spin-wave amplitude is self-limited by the onset of parametric instability. The small values of $\theta_{\max}$ further confirm the consistency of the linear approximation used to describe the DE spin-wave dynamics below the threshold."

3. I am also wondering what is the origin of the SW excitations which form a circular shape. For the case of a 1D grating it seems rather unlikely to have concentric ring excitations to which a circular Fourier pattern would correspond.

   The referee is correct that, at first sight, a circular diffraction pattern is unexpected, since a 1D grating primarily excites spin waves along a single wave-vector direction $q_{\parallel}$. Indeed, at low excitation power, we observe two clear diffraction peaks only along the $q_{\parallel}$ direction. However, above a certain power threshold, the diffraction pattern becomes circular, already pointing at a nonlinear origin, cf. Fig 2e,f.

   We can further neglect direct excitation of magnon-modes with a sizeable $q_{\perp}$ component by the GC and CPW, e.g. due to edge roughness, cf. our response to issue #2 of reviewer 2 and Fig. R2 for more details. Note that also the detection of directly excited BWV modes by the GC or CPW can be ruled out, as they cannot enter the X-ray field of view in the MMM experiment, see Fig. 1a and Extended Data Fig. E4.

   As we further show, this isotropic occupation of magnon modes arises from a nonlinear instability process, predominantly driven by four-magnon scattering, which redistributes the initially excited Damon–Eshbach mode into secondary modes in arbitrary directions.

   In the manuscript, we discuss this as follows:
   L149ff: "*[...] the two anticipated diffraction peaks in the $q_{\parallel}$ direction are accompanied by a distinct elliptical scattering ring (Fig. 2a). This ring clearly indicates the population of magnon modes across all directions of propagation. [...]"*

   We furthermore improve our description in L156ff:
   "Our analysis reveals that the mechanism responsible for populating magnon modes across all directions is a spin-wave parametric resonance driven by four-magnon scattering. In this process, two directly excited DE modes couple to two secondary magnons of arbitrary wave vector $\pm k_{SW}$. The coupling strength is proportional to the population of the DE modes. Parametric excitation becomes possible only when the population of the DE modes reaches a threshold value corresponding to a threshold radio frequency (RF) field (or power) amplitude. The critical field for the parametric excitation–threshold field distribution in momentum space can be derived from the SWM (see Supplementary Information) and is presented in Fig. 2c for an excitation frequency of f_{RF} = 9.00 GHz."

4. I am wondering why in Fig. 2f excitation along the f_rf maximizes at q_perp = 0 while other orders acquire a finite q_perp.

   At the fundamental frequency, f_{RF}, the excitation maximum occurs at $q_{\perp}=0$ corresponding to the most efficient coupling perpendicular to the stripe geometry and exclusively along $q_{\parallel}$. For higher harmonic or fractional-order excitations, the maxima appear at finite $q_{\perp}$, reflecting more complex redistribution of magnon modes. This intriguing feature highlights the rich information content provided by MMM. While

understanding these deep nonlinear interactions in full detail is beyond the scope of the present work, our focus here is to demonstrate the general capabilities of the MMM technique. Currently, we are investigating how these amplitude distributions relate to the underlying nonlinear processes, and a concise interpretation of the fractional parametric modes is not yet established.

We add to the discussion of Fig. 2f in the manuscript (L204ff):
"*The appearance of these modes highlights the deeply nonlinear character of the strongly driven system. While a detailed theoretical understanding of these fractional harmonics is beyond the scope of this study, they underscore the rich information content accessible with MMM and remain a topic for future investigation.*"

5. Why in Fig. 3 the spin-wave dispersions at the multiple f_rf (2rf, 3rf..) seem to have lower frequencies compared to the original? It looks rather counterintuitive.

   This apparent lower frequency arises from the nature of the measurement: MMM directly detects the magnon wave vector rather than its frequency. The higher-order harmonic signals are therefore observed at larger q values. While the excitation frequency of the external drive remains fixed at f_{RF}, the nonlinear system generates harmonic modes at integer multiples of this frequency, which are accompanied by correspondingly larger wave vectors that are detected.

   We agree that this may appear counterintuitive from the perspective of frequency-based detection techniques, and have added a clarification (L227ff):
   "*Note that the higher-harmonic branches appear at lower frequencies than the fundamental because the dispersion in Fig. 3a is shown as a function of the excitation frequency f_{RF}. The actual magnon frequencies, however, obey $f_{SW} = n\, f_{RF}$, and MMM measures the corresponding wave vectors.*"

6. The authors discuss application of the technique to THz waves, obviously referring to AFM systems. Can the authors discuss applicability of their technique to collinear AFM or non-collinear AFM? Would it require higher sensitivity or would one have to switch from XMCD to XMLD, which obviously has orders of magnitude smaller yield?

   We thank the reviewer for addressing this important issue about probing AFM magnons in the THz range. In principle, even for a collinear AFM, there can be non-zero net-magnetization, $\mathbf{M}(t) \neq 0$, in the case of dynamically excited coherent AFM spin waves [Blank et al., *Phys. Rev. Lett.* **131**, 096701 (2023), new Ref. 39]. Depending on the geometry, i.e. magnon-propagation vector $\mathbf{k}_{sw}$ with respect to the direction of the dynamic magnetization $\mathbf{M}(t)$, XMCD can still be a feasible contrast mechanism. In case there is no projection of the dynamic magnetization vector $\mathbf{M}(t)$ onto the X-ray wave vector $\mathbf{k}_i$ possible, XMLD might serve as an alternative contrast mechanism. In both cases, the *magnetic contrast* will be significantly lower than for FM material systems. However, due to the high photon efficiency of the MMM technique, we are confident that our method is sensitive enough to access AFM magnons in future experiments.

   We have extended the sentence about probing AFM magnons in the "Perspective" section (L321ff) and have added Ref. 39 to the manuscript:
   "*It can be extended to detect magnons up to THz frequencies [37, 38] in antiferromagnetic materials by accessing a magnon-driven dynamic net magnetization either by XMCD [39], or depending on the geometry by exploiting X-ray magnetic linear dichroism (XMLD) as a contrast mechanism.*"

Minor questions:

1. Please indicate a coplanar waveguide and the grating coupler in Fig. 1b. It also would be nice to show Fig. 1b in some other place within Fig. 1 otherwise it does not appear in alphabetical order.

   We have now indicated the coplanar waveguide (CPW) and grating coupler (GC) in Panel **b** of Fig. 1. We cannot find a better placement of Panel **b** without significantly increasing the size of the whole figure, or by completely rearranging the sketch in Panel **b**. Accordingly, we would like to keep the arrangement of Panel **b** and order of labels as is.

   [redacted]

   Figure R1: Updated version of Fig. 1, including indicators and labels for the coplanar waveguide (CPW) and grating coupler (GC).

2. I would refrain from saying that the developed model goes beyond LLG which is linear. Generally LLG is nonlinear; it is the linearized version that is linear.

   The referee is correct. We have revised the sentence (L303ff) to:
   "*To interpret this behaviour, we developed a spin-wave model that reformulates the classical Landau–Lifshitz description in terms of spin-wave amplitudes, enabling a clear analysis of the nonlinear interaction terms.*"

Overall, this is a strong and exciting experimental advance. Addressing the major points above (especially the physical origin of the ring, the quantitative estimate of coherent amplitude, and the AFM/XMLD applicability) will significantly increase the manuscript's impact and clarity.

We thank the reviewer for the positive note and hope that we could address all major and minor points above.

Reviewer #2

<u>General report</u>

The authors describe a new X-ray based experimental technique to determine directly a 2D map of spin waves of a given frequency in a thin film, which they call Magnon Momentum Microscopy (MMM). It is based on the scattering of X-rays on linear fronts of spin waves, initially excited directionally using a co-planar wave guide and grating couplers, later converted to all directions above a given power threshold via magnon non-linear

interactions. The technique is clearly novel and provides quick and unlimited access to the 2D map, which can be a very powerful method of thin-film material characterization of spin waves. This alone warrants publication in Nat. Phys. The authors also provide a detailed analysis and modelling of the four-magnon scattering process underlying in their case the non-linear physics required for the technique. This is valuable both from the physical point of view, but also technically to support the analysis of data from the measurements. The manuscript is written with great care and is clear, so are the figures.

We thank the reviewer for pointing out the two main aspects of the work and the positive evaluation of it.

1. I support publication in Nat. Phys. after necessary revision. Detailed comments are provided in the following, some more or less focused, which I advise the authors to consider. Most importantly, I consider that the authors should better explain the generality/limits of the technique, some aspects remaining completely undiscussed. This cannot remain as such while highlighting a new technique: a discussion of applicability is required. A first aspect is the potential application to materials with damping larger than YIG. Does the decay of magnon intensity away from the strip line changes the measurements, their analysis, the occurrence or efficiency of the four-magnon scattering process?

   We agree with the reviewer that a discussion of the applicability of MMM towards other materials than YIG is interesting and relevant. While the (experimental) **analysis** of MMM is generally sample independent, a key distinction must be made between **(i) occurrence or efficiency of the four-magnon scattering process** and the intrinsic **(ii) measurement** capabilities of the MMM technique.

   Regarding (i): The onset or efficiency of nonlinear interactions such as four-magnon scattering, as well as the decay of magnon intensity, are governed by the magnetic properties of the investigated material—such as its damping, anisotropy, and spin-wave dispersion. Stimulated by the reviewer, we discuss the relevance of these parameters for the nonlinear instability process in the Supplementary Information and in the section “Experimental Implications” of the Methods section now in more detail.

   Regarding (ii): The decreased propagation length for materials exhibiting larger damping than YIG, will inevitably reduce the spin-wave amplitude as well as the accessible interaction volume of propagating spin waves with any probe, e.g. photons, electrons, or antenna structures. For the case of MMM, the illuminated sample area can be readily reduced down to diameters of about 1 µm using apertures while maintaining the overall sensitivity and photon flux at state-of-the-art synchrotron radiation facilities. Using dedicated soft-X-ray optics, focus diameters even below 100 nm can be achieved, however implying an increase in beam divergence and hence resulting in a broadening of the diffraction signal in reciprocal space. In analogy to a transmission grating, the diffraction from only a few spin-wave periods also reduces and broadens the diffraction signal in reciprocal space, which can decrease the efficiency of the MMM technique but does not pose a general limitation for the study of nonlinear propagation effects of spin waves.

   Following the reviewer’s remark, we have added the following statement into our “Perspective” (L318ff):
   “*MMM can readily be adapted towards material systems with shorter spin-wave propagation lengths than YIG by reducing the soft-X-ray-magnon interaction volume using smaller X-ray apertures or dedicated focussing schemes down to the sub-µm*

regime.”

2. A second aspect is the cut-off of high-frequency / short wavelengths (both should be discussed as they are linked to different instrumental aspects). While I understand that measurement is possible, I would like to hear more about the generation side. Are there limitations expected arising from the CPW/grating? Is there an impact of strip-line and coupler thickness or side roughness, which would scatter waves already from their emission?

   We emphasize that the MMM **detection** principle is entirely independent of the **generation** scheme. In this first demonstration, a coplanar waveguide (CPW) with a grating coupler (GC) is used as a well-established and controllable source of spin waves, but it is not an essential component of the method and can readily be replaced by other excitation mechanisms, such as optical or THz-driven sources.

   Regarding the **generation** side in our experiment, the CPW and GC provide microwave magnetic fields, which generate oscillating torques on the spins in the underlying YIG film (for detailed information see our Refs. [10,12]). In the linear regime, only the CPW/GC eigenmodes are excited, see Extended Data Fig. 2a. For a given excitation frequency, the excitation strength depends on the matching of wave vectors of the allowed eigenmode in YIG with the CPW/GC modes.

   Edge roughness can, in principle, give rise to weak additional excitation channels by broadening the excitation maxima and introducing small wave-vector components along the long axis of the GC stripes and the CPW. However, the excitation efficiency associated with such statistically distributed inhomogeneities is expected to be negligible compared to that of the deliberately engineered CPW/GC modes. However, to quantify potentially generated wavevectors, we performed a Fourier analysis of the CPW edge roughness, shown in Fig. R2. The resulting wave-vector components are confined to small magnitudes, well below those of interest in the present experiment, and lie entirely within the region blocked by the beamstop. Consequently, any spin waves potentially generated by edge roughness neither contribute significantly to the excitation process nor appear in the detected MMM signal.

   The frequency bandwidth of the CPW depends on its design and dielectric cladding. Supported frequencies could go up to the 100 GHz frequency regime for an appropriately designed CPW. Depending on the generator and integrated cables, we limited our experiments to ≈ 10 GHz.

[redacted]

Figure R2: Supported wave vectors by the edge roughness. Apart from the fact that a major effect of the edges is not expected in terms of generated RF magnetic field and hence magnon excitation, note that the supported wave vectors are small and, hence i) off-resonant for the measurements we show, and ii) would be covered behind the beamstop.

Considering the **detection** limits, MMM itself imposes no intrinsic cut-off at high frequencies, and in terms of wave vector is limited only by the photon wavelength (≈ 1.75 nm in the soft X-ray regime) on the upper side, while the lower limit is defined by the beamstop geometry.

In the “Discussion” section, we address this as follows (L257ff):
“*The upper limit for accessible wave vectors in MMM is ultimately set by the overall diffraction limit, specifically, by the wavelength of the scattered soft-X-ray photons, $\lambda_{ph} \approx 1.75$ nm, corresponding to $2k_{ph} \approx 7200\ \mu m^{-1}$. In our current setup, the maximum detectable wave vector is further limited by the detector geometry, while the beamstop obscures features at $q < 20\ \mu m^{-1}$. However, these setup parameters are readily adaptable to specific experimental requirements.*”

Furthermore, we specify in the Methods section (L382ff):
“*The relative distances between the sample, beamstop, and CCD X-ray detector define the accessible wave vectors in the experiment. Here, we choose the sample-to-CCD distance to z_0 = 260 mm, which defines the scattering signal position on the CCD chip through $d = k_{sw} z_0 / k_i$, with k_{sw} the wave number of the magnon, $k_i = 2\pi E_{ph}/(hc)$ the wave number of the incident soft X-rays. Adjusting these parameters and the beamstop position allows further flexibility regarding the accessibility of magnon wave vectors.*”

3. Finally, the authors claim repeatedly that they address (and the technique is specifically suitable to address) the exchange regime and 100-nm barrier. The exchange regime depends on the type of SW, material, film thickness etc. This claim should be discussed, again contributing to highlight the generality of the claim.

   We thank the reviewer for pointing out this inaccuracy in our manuscript. Indeed, the 100-nm barrier is not **generally** applicable as distinction of the exchange regime. Commonly, for the exchange length of YIG, l_{ex} = 16 nm, the magnon regime for $\lambda_{SW} < 2\pi\ l_{ex} \approx 100$ nm is referred to as *exchange-dominated* in literature. And indeed for the **specific case** of the DE and BWV-modes in our 100-nm-thin YIG film, we observe a clear parabolic shape of the dispersion relation in Fig. 3a, which we assign to this *exchange-dominated* regime.

In order to be clear on this classification, we substitute the phrase "exchange-dominated" when falsely used as a synonym for "sub-100-nm" in our manuscript. Moreover, we changed the relevant part in the introduction as follows (L45ff):
"*As the magnon wavelength decreases, short-range exchange interactions begin to dominate. In the prototypical magnonic material yttrium iron garnet (YIG), this crossover typically occurs for wavelengths below 100 nm. A major challenge in accessing this largely unexplored regime is the ability to reliably excite and detect such short-wavelength modes [5].*"

4. Finally, a personal comment, I leave this also in the hand of the Editor to discuss: I find the term "Microscopy" misleading. The name is in general linked to real-space observation. I find that a name such as "Mapping" would be much more suitable, and the authors can keep the shortcut MMM.

   We are aware that the term microscopy is often associated with real-space imaging. However, the terminology "momentum microscopy" is already well established in other fields—most notably in photoelectron and electron momentum microscopy (see, e.g., Tusche et al., *Ultramicroscopy* 159, 520-529 (2015))—where it similarly refers to direct reciprocal-space imaging with high momentum resolution.

   In this context, the term microscopy appropriately reflects the two-dimensional imaging nature and high-resolution mapping capability of our technique, albeit in momentum rather than real space. The alternative term mapping, while descriptive, could be ambiguous as it does not clearly convey the full imaging character and precision of the method. We therefore believe that Magnon Momentum Microscopy (MMM) most accurately captures both the conceptual and methodological essence of our approach.

Detailed comments

General

5. Vectors are sometimes indicated with arrows (main text), then with boldface (Supplementary information). Please rationalize.
6. Subscripts: sometimes correctly used with slanted for variables and upright for names, such as in M_s. This is the case in the main text. However, is done differently in the Supplementary, s is slanted in M_s.
7. The reading of subscripts combining two different meanings is sometimes difficult, e.g., h_effz (see Eq;2 from Supplementary). First, again z could be slanted. Second, a comma may be used to separate the two subscripts. (check other situations in the entire manuscripts)
8. The usual rule of embedded brackets may be used everywhere for better readability, eg the sentences below Eq.(6) in Supplementary: {…[…(…)…]…} [note: it is suitably used in some places, such as Eq.(8)]

   We have carefully addressed these remarks and standardized the typesetting throughout the manuscript and Supplementary Information. Vector notation, subscripts, and embedded brackets have been made consistent according to the suggested conventions to improve clarity and readability.

Abstract

9. For a non-expert, at this stage the link between exchange interactions and non-linearities will be obscure -> Could you please re-arrange?

10. Mention of two-dimensional: at this stage, may not be clear whether this comes from considering a thin-film (which comes at the end of the sentence), or a specific output of the techniques

    We thank the reviewer for these valuable comments on the clarity of our abstract. We have rewritten the abstract as follows:
    "*Magnons are quantised collective excitations of long-range ordered spins. At nanometre wavelengths, exchange interactions increasingly govern their dynamics, giving rise to a largely unexplored regime of couplings between magnons and other quasiparticles. Yet, detecting such short-wavelength spin waves has remained a key experimental challenge. Here, we introduce magnon momentum microscopy (MMM)– a quasi-elastic, resonant magnetic soft-X-ray scattering technique that directly images magnon populations across two-dimensional momentum space. Owing to its remarkable sensitivity, MMM can capture nonlinear magnon-magnon interactions over large regions of the dispersion plane. Applying MMM to the prototypical magnonic materials yttrium iron garnet (YIG), we uncover a rich variety of previously unobserved nonlinear magnon interactions. With its element specificity, bulk sensitivity, as well as intrinsic access to nanometre-scale wavelengths without frequency limitation, soft-X-ray MMM establishes a powerful and versatile platform for exploring short-wavelength and nonlinear magnonics.*"

Introduction

11. "are considered a promising vehicle to overcome the limitations of predominant CMOS technology in low-power analogue and digital computing as well as signal processing". Please reformulate. There are deep and shared doubts in device players about the broad use of magnonics, owing to the weak efficiency of excitation and detection (voltage-magnon-voltage) conversion. This does not remove any of the excellent achievement in physics in the paper, but being more realistic in the claims for application would be fair.

    Following the reviewer's suggestion, we have reformulated our "Introduction" (L41ff):
    "*Spin waves and their quasi-particles, magnons, provide a versatile platform for exploring alternative concepts for wave-based information processing beyond conventional CMOS technology [1]. In particular, nonlinear magnonics has emerged as a promising domain for realizing computing schemes that exploit the intrinsic nonlinearity of magnon interactions [2–4].*"

12. In the detection schemes, please add the recent demonstration of detection with magnetoresistance by Bailleul and collaborators, which is very promising.

    We have now incorporated this promising recent detection approach based on magnetoresistance, as recently demonstrated by Bailleul and collaborators [Rossi et al., *Sci. Adv.* 11, 33 (2025), new Ref. 17], into the introduction.

13. "Light, however, can be used as a more versatile and non-invasive probe of magnons"
    a. Very short and two arguments, if you wish to keep, please elaborate
    b. Versatile: should be explained why the previous techniques are less"
    c. Non-invasive: should be explained how the previous techniques are invasive.

    We agree with the reviewer, that the *versatility* statement was not well explained. The non-invasive character of light-scattering typically relates to the fact that samples must not be altered, e.g. by growing antenna structures on their surface. In order to focus on the main line of argumentation, we have changed the sentence as follows

(L60ff):
"*Light, however, can be used as a more versatile probe of magnons.* Unlike electronic detection, o*ptical methods do not rely on patterned antennas, contacts, or Hall structures, allowing flexible scattering and spectroscopy geometries under largely unconstrained sample conditions.*"

14. You jump directly from BLS to the required use of X-rays. Could you elaborate on other techniques? There have been nice demonstrations of magnon probing with NV center microscopy or TEM techniques. How they compare should be described to the reader
15. "Across the entire dispersion plane remains a critical challenge" The link with possible limitations of RIXS and STXM have not been made explicit -> Please discuss. Please also explain at this stage why sensitivity matters and why the two techniques mentioned fail.
16. Note: some aspects are covered in the discussion part, but at the stage of the introduction this sounds like an unsupported claim.

We very much acknowledge the reviewer's careful thoughts about our introductory text. However, we do not aim to provide a complete *review* of available short-wavelength magnon detection techniques in the limited space of the "Introduction", and hence we have concentrated on light-based techniques here. However, for completeness, we have added the so far missing information on NV centre and TEM techniques to the "Discussion" part of our manuscript, also introducing three new references [Bertelli et al., *Sci. Adv.* **6**, eabd3556 (2020), Zhou et al., *PNAS* 118, (2021), new Ref. 33 & 34] and [Kepaptsoglou et al., *Nature* 644, (2025), new Ref. 35]:

L279ff: "*Magnon probes other than light are often susceptible to variations in the sample environment or rely on stringent external conditions. For example, the resonance frequency of nitrogen-vacancy (NV) centres shifts in external magnetic fields [33,34], complicating measurements. Likewise, the impressive progress achieved by combining scanning transmission electron microscopy (STEM) and electron energy loss spectroscopy (EELS) to access antiferromagnetic magnons across the full Brillouin zone with meV energy resolution [35] relies on strong magnetic electron lenses, which are difficult to reconcile with most ferromagnetic spin-wave materials.*"

Deliberately, we have chosen not to discuss other experimental techniques in the "Introduction". Instead, we do so in the "Discussion" section, where a direct comparison to our MMM results is more meaningful, and we can further explicitly address the role of sensitivity, see also our response to the next issue. To that end, we intentionally kept the description of the limitations of techniques such as RIXS and STXM rather general in the "Introduction". However, the presence of a *detection gap,* that has been so far inaccessible by these existing X-ray techniques, is well-supported by the immediate observation of previously unseen nonlinear magnon interactions with our MMM technique.

Magnon Momentum Microscopy

17. "Across the entire dispersion plane remains a critical challenge": two things differ between STXM and MMM: the scheme of measurement, and the total flux of X-rays. Please disentangle both, to better understand the underlying mechanism: do we gain mostly because there are more photons here than with a Fresnel lens in STXM?

The improved sensitivity and coverage of MMM compared to STXM originates primarily from the different measurement principle, rather than simply from a higher photon flux. In MMM, the detection occurs directly in reciprocal space via quasi-elastic scattering, where the spin-wave-induced magnetic modulation acts as an effective diffraction grating. This geometry enables the simultaneous detection of all accessible magnon wave vectors within the illuminated area, independent of their real-space location or phase.
In contrast, STXM relies on real-space imaging, requiring spatial scanning and Fourier transformation to access momentum-space information. This inherently limits sensitivity, acquisition speed and, regarding the need for a focussed beam and synchronized operation, also spatial and temporal resolution.

In the manuscript, we address this discussion with:
L269ff: "*STXM has recently demonstrated access to magnons with wavelengths near 100 nm [9, 12]. While powerful in resolving real-space spin dynamics, the sensitivity of STXM to short (and fast) spin-waves is inherently limited by its spatial and temporal resolution, which is dictated by the X-ray optics and pulse duration, respectively.*"
L287ff: *"What sets MMM apart from other approaches is its combination of high sensitivity, rapid acquisition, and direct 2D access to momentum space without frequency limitations–all within a relatively simple experimental setting. These capabilities originate from the underlying scattering process, where spin-wave-induced magnetic modulations form an effective absorption grating. [...] Operating directly in reciprocal space, MMM bypasses the limitations of inefficient X-ray imaging or dispersion optics, with 2D diffraction patterns that directly encode magnon wave vectors and intensities, eliminating the need for complex reconstruction or modelling.*"

FIG.2

18. Second row: the frequency may be mentioned with a label, similar to the first row, to ease the reading of the figure for all frames, without having to read the entire caption.
19. Comment for the caption or related text: so far you have described wavelength (eg for the limitation of existing techniques), now you switch to k vectors. Could you translate these (eg those reported in FIG.2) to wavelength to facilitate the reading by the reader? This conversion is provided in the discussion, which I find too late.

We changed the figure labels according to the suggestion of the reviewer. In the revised manuscript, we now provide the conversion between wave vector and wavelength earlier in the section "Magnon Momentum Microscopy" (L107ff):
"*For spin waves with a well-defined wave vector $\mathbf{k}_{SW}$ (with wavelength $\lambda_{SW}=2\pi/|\mathbf{k}_{SW}|$), magnetic diffraction peaks of +1st and −1st order emerge in the scattering pattern at [....]*"

[redacted]

Figure R3: Updated Fig. 2, with following changes: Added both parameter labels on every graph, power and frequency. The parameter, which stays the same, is depicted in a lighter gray colour. Also, we change the label for the colour scale of the theory graph from "Threshold field (mT)" to "Critical field (mT)" in order to be more explicit on the mechanism.

High-Resolution Mapping of the Spin-Wave Dispersion

20. Traditional methods typically fail to capture the magnonic behaviour in this regime. Please explain in more detail, for the methods mentioned in the intro (plus possibly NV center, TEM). Is it the deep sub-micrometer wavelength that you claim? Or the 2D?

    We discuss the applicability of other *traditional* methods in capturing nanometre-scale magnons in the "Discussion" section (L263ff). Starting in L287ff, we then highlight the specific advantages of MMM. While today, different techniques can access sub-micrometer wavelength magnons, the specific advantages of MMM are the unique combination of direct access to two-dimensional reciprocal space without frequency limitation, and superb signal-to-noise ratio. This together renders MMM as a very powerful and explorative technique, especially for nonlinear magnonics.

Techniques

21. Conversion of dB into OE field in the YIG would be helpful (it is discussed later on in the SWM, because indeed it is the relevant parameter for the threshold of non-linearity).

    We agree with the reviewer that the conversion from RF excitation power (in dBm) to actual Oersted field strength (in mT) provides an important and quantitative link between experiment and theory. To that end, we have performed COMSOL

simulations of the RF field distribution below the coplanar waveguide, see Fig. R4b. We observe that the experimentally generated RF field is consistent with the theoretical threshold field calculations.

[redacted]

Figure R4: Updated Extended Data Fig. E2 including the following changes: a Rescaling to absolute efficiency and correction of sample geometry. **b** New plot of magnetic excitation field distribution below the CPW and GC structure. Red areas depict the CPW and grey areas the GC structure.

In particular, the experimental magnetic excitation field for the experimental RF power of -5.0 dBm that is mainly represented in the manuscript (apart from Fig. 2d-f) is around 1 mT. Comparing this value with the theoretically calculated threshold field (Fig. 2c and Fig. S1) demonstrates that the parametric instability preferentially emerges in regions where the CPW/GC structure provides the strongest RF magnetic field, i.e. where the excitation efficiency is maximal (Fig. R4a).

We can conclude the following:

- At the higher frequencies around 9 GHz (Fig. 2c), the excitation field overcomes the threshold field only in a very narrow band of frequencies and wave vectors (compare Extended Data Figs. E2 and E3). This is reflected by the well-separated diffraction peaks at frequencies not exactly lying on the resonance (see, e.g., Fig. 1), where we are still below the threshold and clearly in the linear regime. Once being exactly on the resonance (Fig. 2a) however, we overcome the threshold field and observe the occurrence of the instability, i.e. the occurrence of the diffraction ring.
- At low frequencies (Fig. S1), the threshold field is well overcome and hence, the nonlinear instability process is triggered consistently in a broad range of frequencies. This is exactly what we observe experimentally.

The “Experimental Implications” subsection in the Method section contains the following paragraph (L479ff):
“*An order-of-magnitude analysis shows that when the CPW and GC efficiency, cf. Extended Data Fig. E2a,* $|\hat{h}^{+}_{k'}| \approx 5\times10^{-3}$*, the threshold field is* $H_{RF,thr} \approx 1$ *mT, whereas for maximum efficiency (*$\approx 2\times10^{-2}$*), the threshold field decreases to* $H_{RF,thr} \approx 0.01$ *mT. These values are consistent with the experiments: the threshold power value at* $f_{RF} =$ *9.00 GHz is* $P_{RF} \approx -5.0$ *dBm (see Fig. 2a). This power corresponds, according to the COMSOL calculations presented in the Extended Data Fig. E2b, to an RF field*

amplitude of 1.3 mT. At lower RF frequencies, f_{RF} = 2.38 GHz, the same level of RF power excites additional higher-order spin-wave modes of frequencies $2f_{RF}$, $3f_{RF}$ and $4f_{RF}$ (see Fig. 2b). The theoretically estimated threshold field (power) is almost two (one) orders of magnitude smaller than the value at f_{RF} = 9.00 GHz. This is due to the higher coupling efficiency of the CPW at lower wave vectors. Therefore, the excitation of higher-order parametric resonances ($n f_{RF}$, n = 2, 3, 4) is consistent with the fact that the RF field (power) significantly exceeds the threshold value."

Reviewer #3

In the manuscript by Wittrock et al., the authors probed the dispersion and nonlinear interaction of magnons by means of quasi-elastic, resonant magnetic soft-X-ray scattering. They reported the excitation of nonlinear magnons by off-resonant excitation frequency, among which the wavelength of magnons can be as short as 100 nm. I find the experimental tools are advanced and impressive. The reported results are interesting and valuable to the community of magnetization dynamics. However, the physics mechanism appears not sufficiently new since the theoretical model is standard, and a careful comparison between the theory and experiment is not performed.
So I find it difficult to recommend the publication of the manuscript of the present form in Nature Physics regarding several main concerns:

We thank the reviewer for acknowledging our experimental advances and valuing our results on nonlinear magnon interactions. We also agree with the reviewer that in the initial version of the manuscript, the comparison between experiment and theory was not sufficiently detailed. Following also the comments of the other reviewers, we have now substantially improved this part of our manuscript, e.g. within the subsection, "Experimental Implications" in the Methods sections.
However, we respectfully disagree with the judgement that our findings do not represent sufficiently new physics just because the "theoretical model is standard". Indeed, the foundation of our theoretical model is the LLG equation and our spin-wave model is inspired by the work of Suhl. However, both in experiment and theory we uncover a so far unseen parametric instability for propagating magnons enabled by the unique capabilities of our MMM technique, which provides direct access to short-wavelength, multidirectional nonlinear processes that could not be resolved with existing techniques so far.

1. The authors addressed ``This magnon momentum microscopy (MMM) can access magnons directly in momentum space with remarkable sensitivity and high photon efficiency up to THz frequencies and down to few-nanometre wavelengths." But in the main text, the excitation frequency is about 10 GHz, and the wavelength of the excited magnon is about 100 nm. So, although the resolution of magnon momentum microscopy could be very sharp, as the authors envision, it is not reported in the present manuscript. In other words, if this resolution can be appropriately reported in this manuscript, it would attract much attention from the magnon community, such that the publication of the manuscript is warranted.

   In the present proof-of-concept demonstration, the **generation** of magnons was indeed limited to wavelengths of about 100 nm in the 10 GHz range. Based on the used microwave CPWs (+ GC), the shortest coherently excited magnon wavelengths can reach the 70–100 nm range–already close to the state-of-the-art limit of spin-wave generation with existing device technology. As discussed in the recent *Magnonics Roadmap* [Flebus et al., *J. Phys.: Condens. Matter* 36, 363501 (2024), Ref. 5], reliable generation of shorter-wavelength magnons remains a major challenge for the field.

The **detection** limit of our MMM technique is, however, only limited by the X-ray wavelength used for scattering (≈ 1.75 nm), which sets an intrinsic momentum sensitivity up to ≈ 7200 μm^{-1} corresponding to magnons with few-nanometre wavelengths. Regarding frequency, MMM is conceptually broadband because it probes only momentum. The technique is therefore fully compatible with THz-frequencies, e.g. for probing antiferromagnetic magnons, see also our response to Referee 1, Comment #6.

To that end, we are firmly convinced that our statement "[...] *magnon momentum microscopy (MMM) can access magnons [...] up to THz frequencies and down to few-nanometre wavelengths*" is well justified, although we were not able to experimentally enter this very exciting new regime, yet.

2. The theoretical model about the nonlinear interaction, including the three-magnon and four-magnon processes, is proper. But there is no careful comparison with the experiment. Although the formula is presented, can the authors use the formula to reproduce the excitation of nonlinear magnons and compare with the experiment?

   First, complementing our earlier clarification to the Reviewer, we would like to emphasize that the parametric excitation observed in our experiment exhibits a degree of novelty compared to the standard Suhl-type processes. In our geometry, the CPW and GC directly excites Damon–Eshbach magnons with finite wave vector, and once their population exceeds a critical value, magnons of different wave vectors–but at the same frequency–are parametrically generated. This differs from the conventional scenario where the uniform (k = 0) mode mediates the instability (from Suhl's seminal work onward, including [Hwang et al., *Sci. Rep.* *11*, 14207 (2021)]). The novelty of the theoretical treatment therefore follows directly from the unconventional parametric pathway revealed by the MMM measurements, even though the mathematical framework of parametric resonance is established.

   To address the reviewer's request for a more quantitative comparison, we have substantially expanded both the main text and the Supplementary Information (see also Reviewer 2's question regarding the dBm-to-Oe conversion #21). Specifically, we derive the minimum RF field required for the onset of the parametric instability (the threshold field) and now compare this prediction directly with the experimental conditions. In addition, we include COMSOL simulations of the RF field generated by the CPW at the sample (Fig R4b, new Extended Data Fig. E2b), enabling an accurate estimate of the actual field amplitudes in the experiment. These considerations, together with the COMSOL-based field estimates, are now discussed in a dedicated Methods subsection, "Experimental Implications", where we outline the connection between the model and the experimental conditions.
   Using these simulated fields as input, the spin-wave model (SWM) quantitatively reproduces the experimentally observed onset of nonlinear magnons, their wave-vector distribution, and the measured power threshold. This agreement holds while explicitly treating both three-magnon and four-magnon scattering channels (Supplementary Information, Eqs. 7–8). These additions demonstrate that the SWM captures the essential nonlinear dynamics responsible for the multidirectional occupation of modes observed in the MMM images.

3. In the theoretical model, can the authors compare the efficiency of three-magnon and four-magnon processes? Which one is dominant under different excitation powers?

   At all excitation powers relevant to our experiment, the four-magnon process

dominates the onset of the observed nonlinear magnon dynamics, which we justify in the Supplementary Information (L678ff) as follows:
"*The linear resonance condition of the DE modes for $\omega_{k'} \approx \omega_{RF}$ maximises the magnitude of the coefficient of the parametric terms and then minimises the threshold for the parametric instability [30]. Moreover, in such a condition, these two types of coefficient scale differently with respect to α. For the yttrium iron garnet (YIG) medium, the value of the damping constant $\alpha \approx 10^{-4}$ is such that the coefficient of the parametric term due to the four-magnon scattering process for $\omega_{k'} \approx \omega_{RF}$ is several orders of magnitude larger than the coefficient of the three-magnon scattering mediated by the RF field. Therefore, this last one can be safely neglected.*"

4. It appears that the theoretical model cannot explain key features of the observation since the authors addressed on page 9 "A complete theoretical framework to explain these harmonics is still lacking and remains a subject for future study." I suggest that the authors compare the theoretical model and observation to clearly state the status of the understanding, i.e., which part can be explained and which part is still a mystery.

We thank the reviewer for the comment. The model reproduces the experimentally observed instability threshold, the multidirectional population of magnons, and the corresponding wave-vector distribution with quantitative agreement (see main text and Supplementary Information). In the revised manuscript, we now perform a more detailed quantitative comparison between the model and the measurements (see former reviewer comments). This establishes that the observed rings arise from a four-magnon parametric process, which is fully accounted for in our analysis.
The present model does not describe all features observed at higher excitation powers. In particular, the appearance of (sub-)harmonics and the detailed azimuthal redistribution of magnon intensities are not captured within the current framework, as these likely involve additional nonlinear mechanisms beyond the standard three- and four-magnon interactions included here. A complete theoretical description of these phenomena will require further developments and is beyond the scope of the present work, whose primary goal is to introduce MMM as a new experimental technique for momentum-resolved magnon imaging. The main purpose of our theoretical model is to capture the essential nonlinear processes underlying the formation of the elliptical magnon rings and to verify the magnetic origin of the MMM signals.

To make this discussion with the reviewer explicit in the manuscript, we have added the following clarification to the discussion of Fig. 2f (L201ff):
"*As the power increases, a fundamental dispersion ring forms, followed by additional parametric processes that generate spin waves at fractional harmonics of the fundamental frequency ($f_{SW} = m/8\ f_{RF}$, with m = 3 to 14, except for m = 4 and 10). The appearance of these modes highlights the deeply nonlinear character of the strongly driven system. While a detailed theoretical understanding of these fractional harmonics is beyond the scope of this study, they underscore the rich information content accessible with MMM and remain a topic for future investigation.*"
